# Modelling forest carbon stock changes as affected by harvest and natural disturbances. I. Comparison with countries’ estimates for forest management

**DOI:** 10.1186/s13021-016-0047-8

**Published:** 2016-05-23

**Authors:** Roberto Pilli, Giacomo Grassi, Werner A. Kurz, Raúl Abad Viñas, Nuria Hue Guerrero

**Affiliations:** 1 European Commission, Joint Research Centre, Institute for Environment and Sustainability, Via E. Fermi 2749, 21027 Ispra, VA Italy; 2 Natural Resources Canada, Canadian Forest Service, Victoria, BC V8Z 1M5 Canada

**Keywords:** Net CO_2_ emissions, Greenhouse gas inventories, European countries, Carbon Budget Model, Forest management, Harvest, Natural disturbances

## Abstract

**Background:**

According to the post-2012 rules under the Kyoto protocol, developed countries that are signatories to the protocol have to estimate and report the greenhouse gas (GHG) emissions and removals from forest management (FM), with the option to exclude the emissions associated to natural disturbances, following the Intergovernmental Panel on Climate Change (IPCC) guidelines. To increase confidence in GHG estimates, the IPCC recommends performing verification activities, i.e. comparing country data with independent estimates. However, countries currently conduct relatively few verification efforts. The aim of this study is to implement a consistent methodological approach using the Carbon Budget Model (CBM) to estimate the net CO_2_ emissions from FM in 26 European Union (EU) countries for the period 2000–2012, including the impacts of natural disturbances. We validated our results against a totally independent case study and then we compared the CBM results with the data reported by countries in their 2014 Greenhouse Gas Inventories (GHGIs) submitted to the United Nations Framework Convention on Climate Change (UNFCCC).

**Results:**

The match between the CBM results and the GHGIs was good in nine countries (i.e. the average of our results is within ±25 % compared to the GHGI and the correlation between CBM and GHGI is significant at P < 0.05) and partially good in ten countries. When the comparison was not satisfactory, in most cases we were able to identify possible reasons for these discrepancies, including: (1) a different representation of the interannual variability, e.g. where the GHGIs used the stock-change approach; (2) different assumptions for non-biomass pools, and for CO_2_ emissions from fires and harvest residues. In few cases, further analysis will be needed to identify any possible inappropriate data used by the CBM or problems in the GHGI. Finally, the frequent updates to data and methods used by countries to prepare GHGI makes the implementation of a consistent modeling methodology challenging.

**Conclusions:**

This study indicates opportunities to use the CBM as tool to assist countries in estimating forest carbon dynamics, including the impact of natural disturbances, and to verify the country GHGIs at the EU level, consistent with the IPCC guidelines. A systematic comparison of the CBM with the GHGIs will certainly require additional efforts—including close cooperation between modelers and country experts. This approach should be seen as a necessary step in the process of continuous improvement of GHGIs, because it may help in identifying possible errors and ultimately in building confidence in the estimates reported by the countries.

**Electronic supplementary material:**

The online version of this article (doi:10.1186/s13021-016-0047-8) contains supplementary material, which is available to authorized users.

## Background

The United Nations Framework Convention on Climate Change (UNFCCC) and its Kyoto protocol (KP) recognize the role of forests in mitigating climate change. Emissions and removals from forests are included in the greenhouse gas inventories (GHGIs) submitted annually by developed countries to the UNFCCC, and typically represent by far the most important component of the “Land use, Land-use Change and Forestry” (LULUCF) sector. Inventories should follow the methodological guidance prepared by the Intergovernmental Panel on Climate Change (IPCC).

The forests in the European Union (EU, including 28 countries) cover about 165 Mha, they increased by about 4 % since 1990 and about 83 % of this area is available for wood supply [1]. According to the EU GHGI, between 1990 and 2012 the average annual sink of EU forests was about 435 Tg CO_2eq_. year^−1^, or about 9 % of the EU total emissions in the same period [2].

For the first commitment period of the KP (CP1, 2008–2012) the accounting of emissions and removals was mandatory for afforestation/reforestation and deforestation (AR and D, i.e. forest land-use changes since 1990) and voluntary for forest management (FM, i.e. forest existing before 1990). For the second commitment period of the KP (CP2, 2013–2020), significant revisions of accounting rules were agreed [3], as reflected in the latest IPCC guidance [4]. The major changes for the forest sector are: (1) the accounting of FM is now mandatory; (2) the FM accounting shall include the carbon (C) stock changes in the harvested wood products (HWP) pool; and (3) emissions and subsequent removals from natural disturbances may be excluded from the accounting. These changes represent new challenges for countries when developing their GHGIs.

Since the GHGIs represent the basis for assessing the effectiveness of any national climate policy, building confidence in their accuracy is of key importance for advancing the international efforts to mitigate climate change. While the GHGIs are subject to an UNFCCC expert review process, which aims to assess the adherence of GHGIs to IPCC guidance in terms of general reporting principles,^1^ this expert review does not include an independent verification of the reported estimates. The verification activities should be performed by each country, as part of the process of improving the GHGI and build confidence in its reliability [5]. However, at the EU level, few countries report efforts or results of verification for the LULUCF sector [2]. In most cases, a real verification is very difficult due to the lack of truly independent and comparable data. For example, since GHGIs cover only emissions and removals from managed lands, an inherent mismatch exists for LULUCF between GHGIs and estimates based on process studies or atmospheric methods [5]. As alternative, a largely independent comparison may be conducted between GHGIs and large-scale models (e.g. [6, 7]) that use data from National Forest Inventories (NFIs). While not a fully-independent verification, such comparisons may be very useful in building confidence in GHGI estimates and trends, improving scientific knowledge and identifying potential problems. The major challenges for this approach are to implement a model capable to reflect the latest IPCC guidance (e.g., including the HWP and natural disturbances, [4]) and to use adequate input data from the countries.

The general aim of this study is to implement a consistent methodological approach using an internationally well established forest carbon budget model to simulate for the period 2000–2012 the impacts of harvest and salvage logging, natural disturbances and land-use changes on forest CO_2_ emissions and removals in all EU countries for which adequate information was available (26 countries out of 28). To this aim, the Carbon Budget Model (CBM) developed by the Canadian Forest Service [8] was used, as part of a broader effort for a comprehensive modelling framework for the forest sector [9]. The model was applied and validated at regional and national scales in Canada [10, 11] and Russia [12]. Furthermore, the CBM was successfully adapted to specific forest management conditions in Europe (e.g. uneven-aged forests, [13]), validated at regional level [14] and applied in one country case to estimate the C balance for FM [13] and AR [15].

Specific objectives of this paper are: (1) to validate the CBM against totally independent data available at the country level (for one case study) and to provide a detailed description of four representative country cases; (2) to compare FM estimates from CBM with each country’s GHGI in terms of trends and levels of net CO_2_ emissions for each forest C pools (living biomass, dead organic matter (DOM) and mineral soil); (3) to analyze how the main drivers affecting the living biomass (harvest and natural disturbances, including major storms and fires) affect the estimates obtained with the CBM and the GHGIs.

A companion paper [16] provides an analysis of the CBM results at the aggregate EU level, including net CO_2_ emissions in the HWP pool and the impacts of forest-land use changes.

## Results

### Model validation

**Fig. 1:**
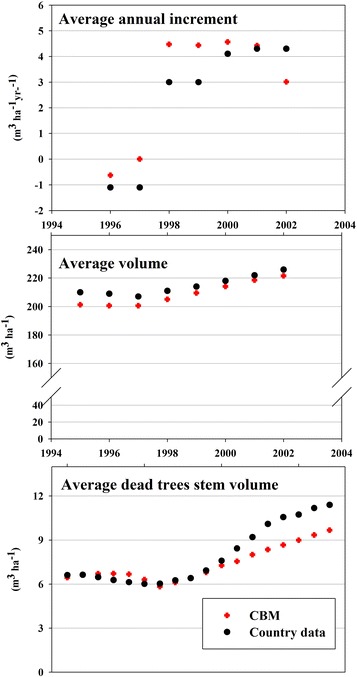
Comparison between the net annual increment (m^3^ ha^−1^ year^−1^), the average volume (m^3^ ha^−1^), and the average dead stems volume (m^3^ ha^−1^) estimated by CBM and reported by Lithuanian NIR [17], based: (1) for volume and increment data, on a specific study on the “Forest Land Changes in Lithuania between 1990 and 2011” and on NFI permanent sample plots and (2) for the dead stem volume, on a specific analysis until 2001 and on NFI permanent sample plots from 2002 to 2012

To validate our model’s results with independent data sources (i.e., not used as input data by CBM), we first compared the mean annual increment and the average volume estimated by CBM (based on the equations applied by the model during the run and the values of merchantable C stock provided for each species) with the additional, independent data reported in the Lithuanian GHGI (Fig. 1). A further comparison is made with the dead tree stems volume estimated by CBM and the values reported by NIR, based on a specific analysis until 2001 and on NFI permanent sample plots from 2002 to 2012.

**Fig. 2:**
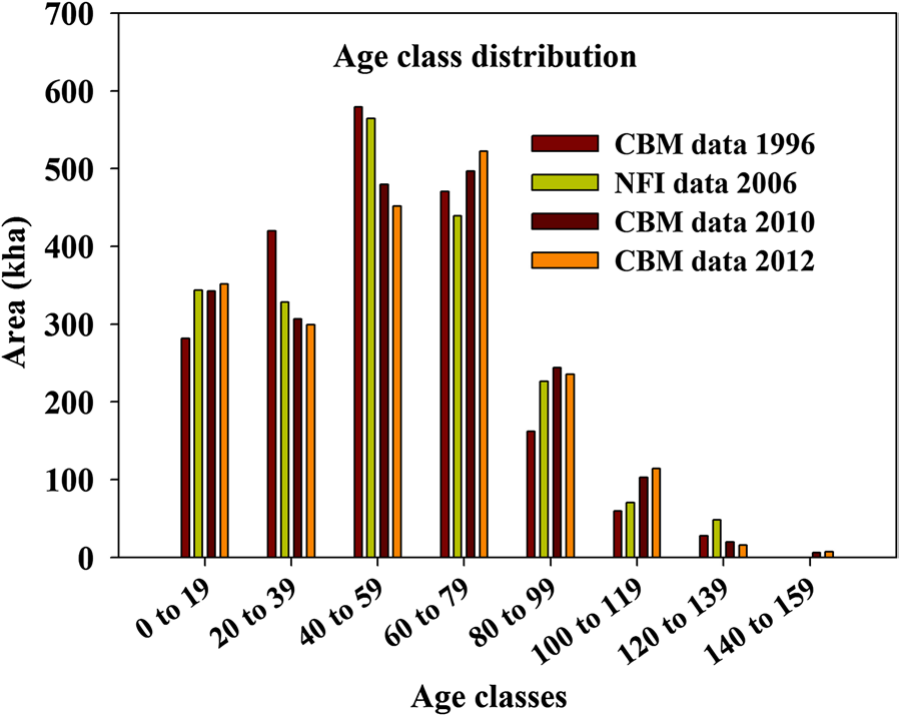
Age class evolution provided by the CBM model from 1996 to 2012, including the effect of deforestation; the original NFI age class distribution (assigned to 2006) is also reported

The model’s results can be further compared with other information for Lithuania, not fully independent of the input data used by CBM, because derived by the same data sources (i.e., NFI). Figure 2 reports the age class evolution estimated by CBM between 1996 and 2012, compared with the original age class distribution reported by NFI 2004–2008 (attributed to 2006).

**Fig. 3:**
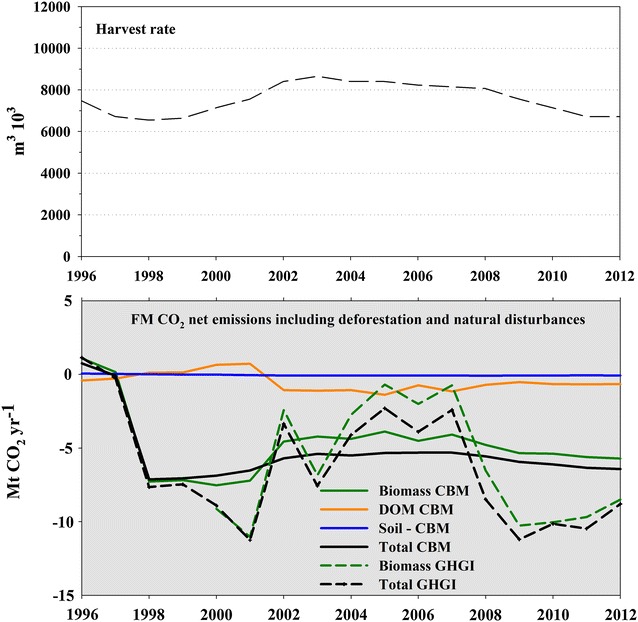
The* upper panel* reports the harvest rate (m^3^ 10^3^) applied to Lithuania by our study; the* lower panel* reports the net CO_2_ emissions estimated by CBM (Mt CO_2_ year^−1^), further distinguished between living biomass, DOM (dead wood + litter) and soil pools and a comparison with the net CO_2_ emissions reported by the country for the land use category FL–FL (Lithuania, [18]), assumed as a proxy of the FM area, for those counties where FM had not been elected during the first KP commitment period)

In Fig. 3 (lower panel), the net CO_2_ emissions estimated by CBM (further distinguished between living biomass, DOM and soil pools) are compared with the net emissions reported by the country’s GHGI (in 2014) for the land use category forest land remaining forest land (FL–FL) (Lithuania, [18]). For Lithuania, our simulation starts in 1996 when, due to the effect of insect disturbances (see the Additional file: 1 for further details), we estimated a C source, consistent with the data reported by the country, and with the mean annual volume increment reported in Fig. 1. From 1997 to 2001, the model estimates an increasing C sink, mainly due to a reduction of the regular harvest, because of the salvage of logging residues. From 2002, the C sink decreases due to the increasing harvest demand (reported in the upper panel of Fig. 3) and, after 2007, the sink again increases following the decreasing amount of harvest. Further inter-annual variations are due to the effect of storms (in 2005 and 2007, according to the information by NIR), while the effect of fires is negligible. The interannual variability in net CO_2_ emissions reported in the GHGI is considerably larger than estimated in the CBM. From 2007, the forest C sink reported by the country strongly increases, from −1.9 Mt CO_2_ in 2007 to −8.0 Mt CO_2_ in 2008 (i.e., +300 %), even if the total harvest demand decreases only slightly, from about 8.1 to 8.0 million m^3^ (i.e., −1.2 %). This reduction was not observed in CBM results, which report only a slightly increase in the C sink between 2007 and 2012, which is consistent with the decreasing harvest rates.

### CBM results vs country GHGIs

**Fig. 4:**
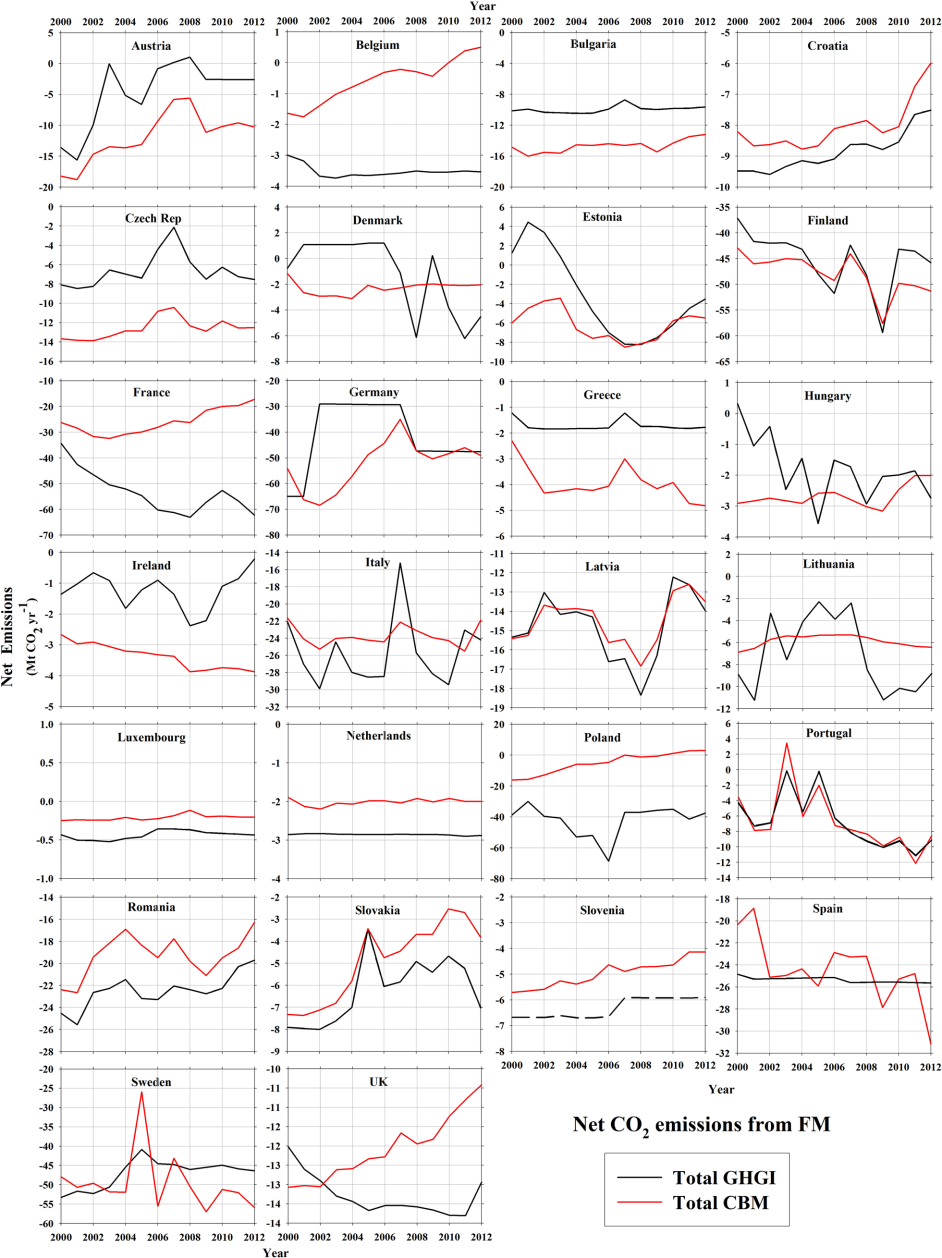
Comparison between the net CO_2_ emissions from FM reported by the countries for the period 2000–2012 (in the 2014 GHGIs, [18]) and the CBM estimates. Data are reported from an atmospheric perspective, where negative values represent a sink (CO_2_ removals) and positive values a source (CO_2_ emissions)

Net CO_2_ emission estimates for the period 2000–2012 as estimated using the CBM and as reported by 26 EU countries in their 2014 GHGI show a wide range of patterns (Fig. 4). Data are for the area subject to FM^2^ and are reported from an atmospheric perspective, where negative values represent a sink (CO_2_ removals) and positive values a source (CO_2_ emissions). Results focus on CO_2_ and exclude organic soils. Non-CO_2_ emissions (CH_4_, N_2_O) from forests may be important only for specific countries, in case of drained organic soils (not included in this paper) and in case of fires, for which we report results in terms of CO_2_-eq for Portugal in the Additional file: 1.

The aggregated results at the EU level and including the harvested wood products (HWP) pool, afforestation/reforestation and deforestation, will be reported in a companion paper [16].

**Fig. 5:**
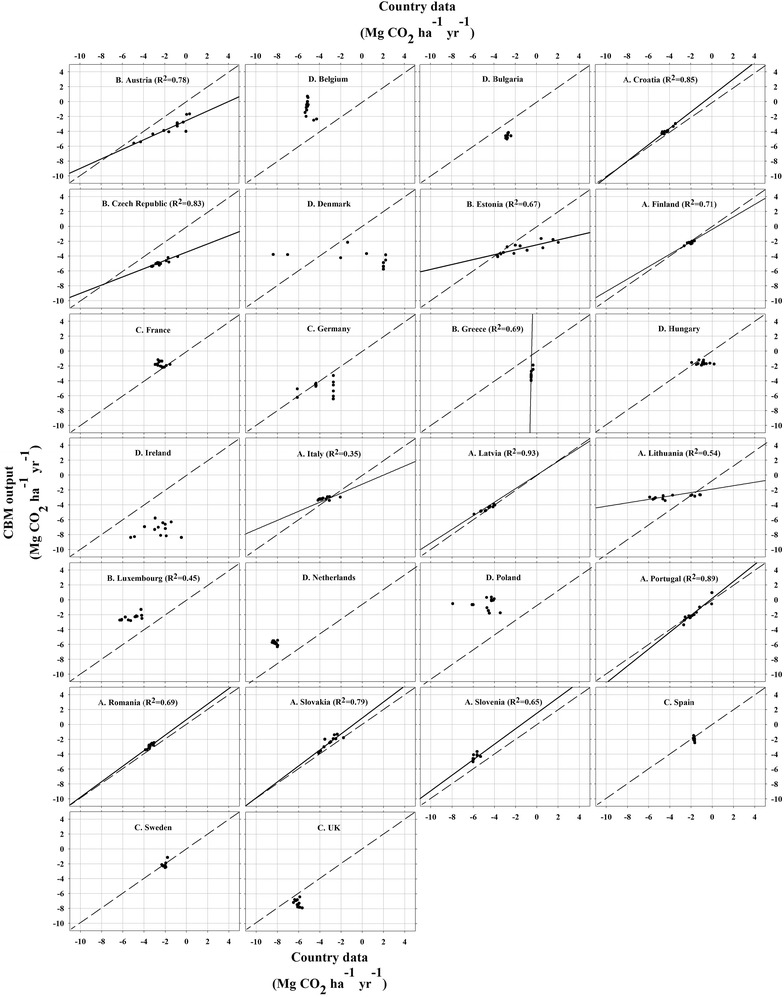
Comparison between the net CO_2_ emissions from FM (living biomass, DOM and mineral soil) as estimated by the CBM and reported in the countries’ GHGIs. Each* point* represents one year for the period 2000–2012, as shown in Fig. 4. The *dashed line* is the 1:1 line. The *solid line* is the regression line, shown where the correlation between the CBM and GHGIs was statistically significant (P < 0.05)

The results obtained from the GHGI and the CBM for these 26 countries can be assessed in terms of *level* and *trend*. For the *level*, we consider the match between CBM and each GHGI as “good” if the average net emission of CBM for the period 2000-2012 (Fig. 4) is within ± 25 %^3^ compared to the GHGI. For the *trend*, Fig. 5 shows the correlation between CBM and each GHGI. In this case, we consider the match between CBM and each GHGI as “good” if the correlation is significant at P < 0.05.

Based on the match between CBM and GHGIs, in terms of *level* and *trend*, and on data reported in Figs. 4, 5, four different groups of countries may be distinguished:

A. Countries where CBM estimates and country data show a good match both in the *trend* and the *level*. This group includes nine countries: Croatia, Finland, Italy, Latvia, Lithuania, Portugal, Romania, Slovakia and Slovenia.
B. Countries where there is a good match in the *trend* but not in the *level*. This group includes five countries: Austria, Czech Republic, Estonia, Greece and Luxembourg,
C. Countries where there is a good match in the *level* but not in the *trend*. This group includes five countries: France, Germany, Spain, Sweden and United Kingdom.
D. Countries where the match is not good for the *level* and for the *trend*. This group includes seven countries: Belgium, Bulgaria, Denmark, Hungary, Ireland, Netherlands and Poland.

**Fig. 6:**
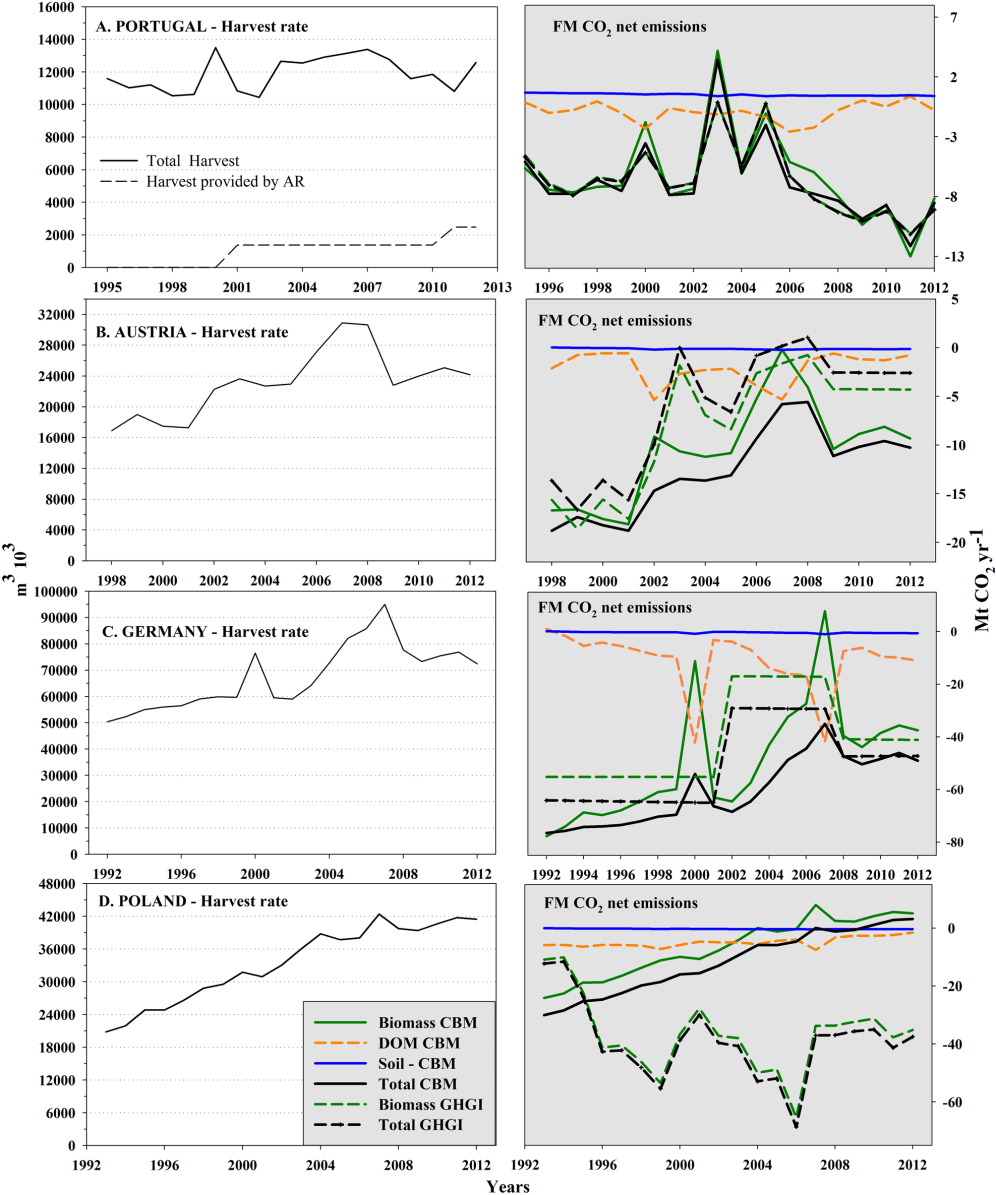
Harvest rate (on the *left panels*, in m^3^ 10^3^) and the main output provided by CBM for four representative case studies (Austria, Germany, Poland and Portugal). For each country we report the net CO_2_ emissions estimated by CBM (Mt CO_2_ year^−1^, *right panel*), further distinguished between living biomass, DOM (dead wood + litter) and soil pools and a comparison with the biomass and the total net emissions reported by each country. When available, FM country data from the KP-CRF tables was used for 2008–2012 (i.e., if FM had been elected during the first KP commitment period); alternatively, country data were taken from the Convention CRF tables using ‘forest land remaining forest land’ (FL remaining FL) [18]. For Portugal, the amount of harvest provided by afforestation (AR) is also reported (panel **a**, *left* panel)

Figure 6 illustrates in more detail the results from four country cases (Discussed in “Country case studies” section.), each representative of the four groups above: Portugal (A), Austria (B), Germany (C) and Poland (D).

## Discussion

### Model evaluation

We implemented a consistent methodological approach to 26 EU countries, using the Carbon Budget Model to estimate the net CO_2_ emissions for the period 2000–2012. To evaluate the capacity of the CBM to reproduce country data, our results can be compared with different data sources available at the country level, such as the age-class distribution reported by the NFI and the net CO_2_ emissions reported by the country’s GHGI. As expected, the comparison between the model results and the country GHGIs showed good agreements in level and trend for some countries and partially good for other countries. When the comparison was not satisfactory, in most cases we can identify possible reasons for these discrepancies. In many cases, however, these data are not fully independent from the NFI input data used by CBM. Where additional information is provided by independent studies (i.e., different datasets, not used by CBM), an independent validation of the model’s output is possible. This is the case of Lithuania, where additional information on the living biomass increment, biomass volume and on the dead tree stem volume is available [17]. We select these parameters because increment is one of the main drivers affecting biomass growth estimated by the CBM, initial volume is the main parameter affecting biomass C stock at the beginning of the simulation and dead tree stem volume is the second major C pool with C stock changes over time, for the majority of the European countries (this is often due to the effect of natural disturbances). For Lithuania we verified that our estimates are consistent with these independent data sources. Of course, as highlighted by Vanclacy and Skovsgaard [19], the effective evaluation of a forest growth model is a complex and ongoing process, that could include additional independent validations performed at the regional level [14], sensitivity analysis of the main input data, and further comparison of our estimates with other data sources, including the country-specific GHGI data (see also other comparisons reported in the Additional file:1 for additional case studies). For Lithuania, the country’s GHGI reports some peaks between 2000 and 2008, not highlighted by our model (see Fig. 3, lower panel). Apart from different assumptions on the area affected by storms and on the salvage of logging residues (we considered three main disturbance events, described in details in the Additional file: 1), these differences may be even due to the interannual statistical variability associated to the stock-change approach, that can exacerbate the real variability of the C stock changes [17]. Despite this different representation of the interannual variability, the overall match between the CBM results and the Lithuania’s GHGI is good, i.e. the average of our results is within ±25 % compared to the GHGI and the correlation between CBM and GHGI is significant at P < 0.05.

### Country case studies

Based on comparisons of both level and trends in CO_2_ emission estimates obtained from the CBM and the country GHGIs we partitioned the 26 countries into four groups, and we discuss one representative country for each group.

For Portugal, such as for other eight countries (Group A), the CBM estimates and country data show a good match both in the *trend* and the *level*. The C balance of this country is strongly affected by inter-annual variations in harvest demand and direct fire emissions (Additional file: 1 for further details). The total C sink estimated by CBM is slightly lower than the reported values but it has the same trend and the differences decrease with time (in 2011 we reported the same values). These differences may be due to the relative amount of harvest provided by Eucalyptus plantations accounted as AR (from less than 15 % of the total amount of harvest in 2002 to about 25 % in 2011, as highlighted in the harvest’s panel of Fig. 6, panels A). As expected, DOM and living biomass pools showed an opposing pattern: when fires kill trees and decrease the biomass C stock, we observe an increase in DOM C pools (i.e., the transfer of C to dead wood and litter add more C than is lost from these pools during the fire).

For Austria, such as for other four countries included in Group B, the CBM estimates show a good match in the *trend* but not in the *level*. In these cases, the different level may be caused by a number of reasons (different conversion factors, different input data, etc.). In the case of Austria, the CBM simulation represents the impact of various natural disturbances. The biomass C balance estimated by CBM (Fig. 6, panels B) follows the same trend that is reported by the country until 2006 and it is strongly affected by the inter-annual variations due to the impact of storms and insect attacks. Indeed, we highlighted a significant statistical correlation (*r* = 0.77) between the total C sink reported by the country and the amount of volume damaged by bark beetle between 1998 and 2007 (see Additional file: 1 for further details). In 2003 and 2005 however, the total C sink reported by the country is considerably lower than our estimates. This may be due to different assumptions about the effect of natural disturbances in specific years. Overall, the biomass C sink estimated by CBM is consistent with the reported trend (Fig. 6). As expected, the DOM C sink has an opposite trend compared with the living biomass. Storms and insect attacks moved C from the living biomass to the dead wood pool and, subsequently salvage logging moved C to the products pool. Yet this impact was not reported by the country’s data (which report a stable C source from DOM pools, equal on average to + 1.8 Mt CO_2_ year^−1^ between 1998 and 2012). This may also explain the differences between our estimates on the total C sink and the values estimated by country: for example, in the CBM in 2007 a strong reduction of the living biomass pools due to a storm is compensated by a corresponding increase in the DOM pools. After 2008, due to different assumptions about the average amount of harvest and about the effect of natural disturbances (country’s data report a constant amount of harvest equal to about 25 million m^3^ from 2009 to 2012) our estimates are not comparable with the country because we used different harvest rates.

Germany (Group C, including five countries), represents an example where there is a good agreement in the *level* but not in the *trend*. We use it to illustrate the difference between the stock-change approach used in the GHGI and the gain-loss method used in the CBM. This methodological difference has a strong impact on the inter-annual variability of estimates as affected by harvest and natural disturbances. Overall, the total C sink estimated by CBM follows the same trend provided by the country (Fig. 6, plot C), even if the correlation is not significant at P < 0.05 (i.e., the threshold considered by our study). Due to the stock-change approach, the national sink estimates report three annual values, each applied to the inventory period over which observed stock changes have been annualized [20]. Compared to the reported values, our estimates show a larger inter-annual variability (in particular for the living biomass and DOM pools) due to the storms that occurred in December 1999 (assumed as 2000) and 2007. As expected, the CBM reports opposite trends in the biomass and DOM pools due to the transfer of C from living biomass to the dead wood pool. From 2008 to 2012, our estimates are fully consistent with the data reported by Germany. Further details on natural disturbances and the evolution of the age-class distribution are reported in the Additional file: 1.

For Poland, such as for other 6 countries included in Group D, the estimates differ significantly for both the *trend* and the *level*, for reasons that will require further analysis. For this country, the CBM estimates a decreasing C sink, consistent with a strong increase of the total amount of harvest reported by FAO statistics (see the left panel of Fig. 6, panel D). In contrast, Poland reports an increasing sink with increasing harvest rate. According to our estimates, the DOM pool (not reported by the country) is a C sink, because of the amount of residues left after harvest (i.e., moved from living biomass to DOM). In addition storms in 1999 and 2007 also moved C from living biomass to the dead wood pool (see Additional file: 1 for further details).

### CBM results *vs.* GHGIs: impact of carbon pools coverage, harvest and natural disturbances

A first, potentially relevant factor, to be considered when comparing the CBM results with the GHGIs, is the inclusion of C pools. The CBM includes all forest C pools (living biomass, DOM and mineral soils) for all countries, but DOM and soil pools are not reported in some GHGIs. While all 26 countries report living biomass, seven do not report DOM and 14 do not report mineral soils [2]. The mineral soil is in most cases neither a large sink or source (in the CBM, and in the GHGIs). In contrast, the CBM estimates of net CO_2_ emissions for DOM pools can be large when natural disturbances occur. Nevertheless, differences in the reported C pools help to explain the observed differences between the CBM and the GHGIs in only a few cases (e.g., Czech Republic, Romania, Slovakia and Slovenia). It is therefore necessary to extend the analysis to the impact of the main drivers of net CO_2_ emissions, i.e. harvest and natural disturbances as indicated in both the CBM and GHGIs results.

**Fig. 7:**
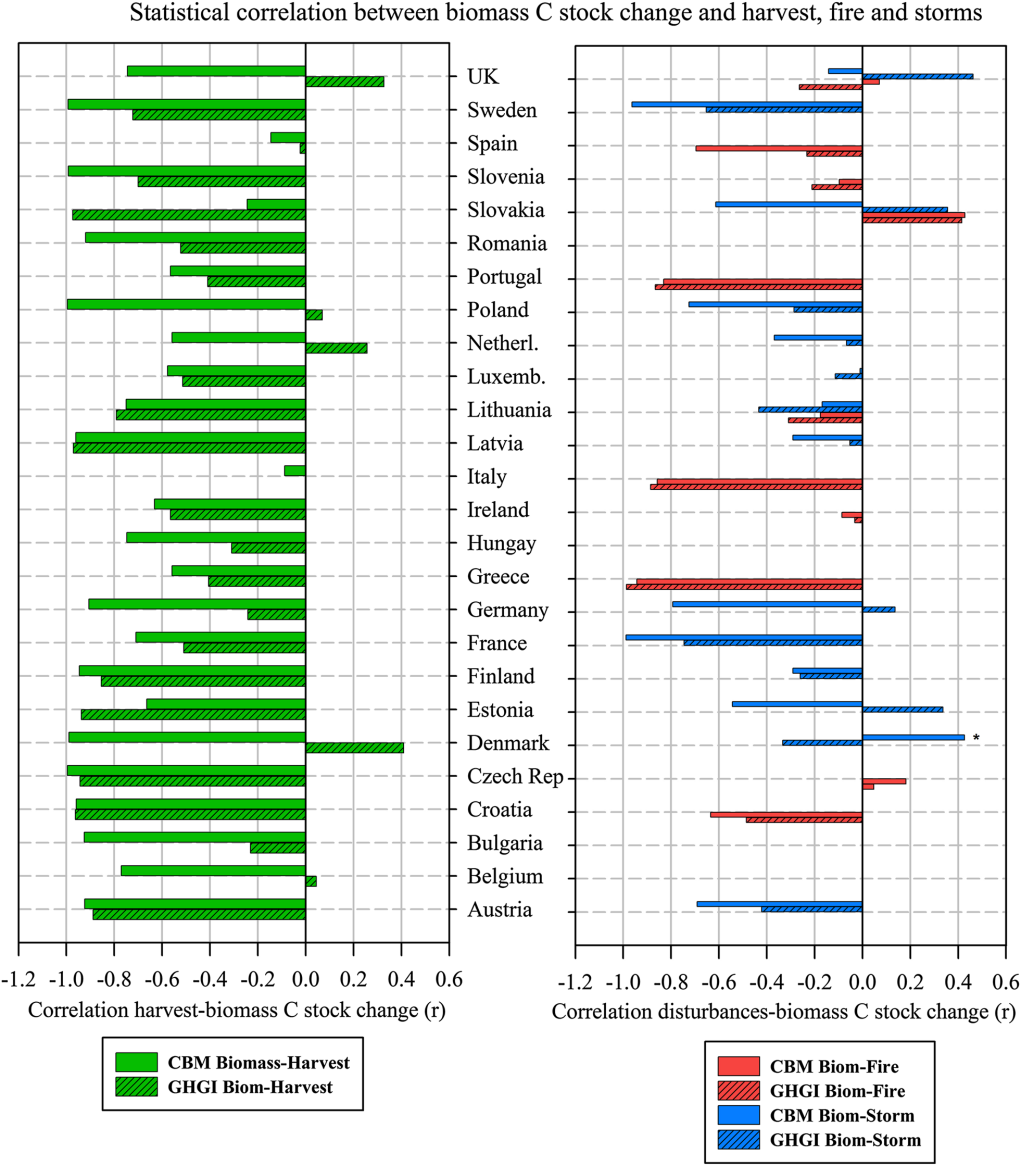
Correlation (*r*) between the biomass net CO_2_ emissions (estimated by CBM and reported in the countries’ GHGIs) and the total amount of harvest (m^3^ year^−1^; *left panel*) and the area affected by fires and storms or insect attacks (ha year^−1^, *right panel*). In some cases, the correlation is not statistically significant (*), due to the number of available observations

The net CO_2_ emissions from living biomass are generally correlated with the three main drivers: harvest rate, area affected by fires and area affected by storms in both the estimates from the CBM and the GHGI (Fig. 7).

The correlations shown in Fig. 7, demonstrate that for 21 out of 26 countries there is, as expected, a clear negative correlation (generally with r < −0.5) for both CBM and the countries’ GHGIs, i.e. more harvest decreases the biomass sink (see for example, Croatia, Finland, Latvia, Lithuania, Portugal, Romania and Slovenia).

Within this group, in three cases (Germany, Estonia, and Slovakia) the correlation between biomass net emissions and the area affected by disturbances is negative for CBM and, surprisingly, is positive for the countries. For Estonia and Slovakia the differences may be due to different assumptions on the effect of storms on the living biomass or DOM pools (i.e., the amount of biomass moved to DOM or removed with salvage logging). For Germany, the main reason appears to be the stock-change approach applied to consecutive NFIs [20]: this approach does not capture the inter-annual variations within a measurement period caused by natural disturbances.

In other cases, despite both the CBM and the GHGIs showing a similar (negative) correlation between the biomass net CO_2_ emissions and both harvest and natural disturbances, overall the match between modelled trends and the GHGI is not good (see Fig. 4). For Austria, the main difference lies in different assumptions about the mineral soil pool (which is a source in the GHGI) and partly about the DOM, since the match between the CBM and the GHGI is good for the living biomass. For France, the discrepancy between the CBM and the GHGI requires further investigation, especially with regard to possible differences about harvest assumptions and increment. For Greece, the total FM sink estimated by the CBM is higher than the values reported by the country, but different assumptions on the effect of fires (for example on the amount of biomass burned and the distribution of fires between the FM area and the unmanaged forest area) could explain some these differences. The FM sink reported by Hungary is considerably lower than our estimate and it shows a higher inter-annual variability, for reasons that are not yet understood. For Ireland, the total sink reported by the GHGI has the opposite trend (i.e., a decreasing C sink) compared with our estimates. Ireland did not elect FM under the CP1 therefore the values reported for this country were derived from the FL remaining FL land use category and a certain amount of harvest is certainly provided by afforestation [21]; this may explain the differences observed. The sink reported by Luxembourg is considerably higher than our estimates and does not seem compatible with the harvest rate applied by CBM. The FM sink estimated by CBM for Spain is overall quite similar to the country GHGI; the main difference is that CBM shows inter-annual variability due to fires and harvest rates, while the stock-change approach implemented by Spain’s GHGI masks this variability [22]. Emissions from forest fires estimated by CBM are generally lower than the CO_2_ emissions reported by Spain. This is probably due to different assumptions on the amount of biomass and DOM burned. For Sweden, the differences detected on the trend may be due to the effect of storms (above all in 1999 and 2005) and an overestimate on the biomass C stock by CBM. A special case is the lack of any correlation for Italy, where the main driver of the inter-annual variability in biomass net CO_2_ emissions is clearly fire (r < −0.80), as also highlighted by [13].

For 5 out of 26 countries (Belgium, Denmark, the Netherlands, Poland and UK), the correlation between biomass net CO_2_ emissions and harvest rate is negative (r < 0) for CBM and, surprisingly, is positive (r > 0) for the country GHGI. In principle, this discrepancy may be explained by three reasons. First, the harvest rate applied by our study is different from the harvest reported by the country in its GHGI; even if we always tried to be consistent with the harvest reported by countries, some differences may exist due to inconsistency between different data sources (e.g. see [23]). Second, other factors (e.g. natural disturbances or rapid changes in net increment not included in our study) are a more important driver of biomass net CO_2_ emissions compared to harvest; although this case does not seems very likely, it cannot be totally ruled out. Third, the estimation method used by the country in its GHGI masks the effect of harvest on the biomass carbon stock change.

For both the Netherlands and UK, a good match in both the trend and the level existed between CBM and the 2013 GHGIs, suggesting that some recent changes (in input data and/or method) were implemented for the 2014 GHGI. For Denmark, although the known most relevant storms (1999/2000 and 2005) were considered by CBM, the overall correlation between CBM and Denmark GHGI is poor (see again Fig. 4). This could potentially be explained by the method used by Denmark, where a stock-change approach is implemented every year based on the information collected annually from the NFI [24]. It is possible that the interannual statistical variability of data associated to this approach overrides all the other factors considered and exacerbates the real interannual variability of C stock changes. For Belgium and Poland further analysis is needed, to explain the observed differences between our results and the country’s estimates. These may be due to the lack of data or to some incorrect assumption on the input data (i.e., the harvest).

### Summary of the main differences between CBM and countries’ estimates

Since the CBM and the GHGIs typically share most of the basic input data (e.g., forest area, timber volume and net increment, taken from the NFIs), we briefly discuss the level of independence of input data. Forest area will be strongly correlated between the CBM and the GHGIs, because we used whenever possible the area used by the GHGI for FM (or, for countries that did not elect FM, for FL remaining FL).

The methods used to estimate the emissions/removals per unit of area—typically the major source of uncertainty of GHGIs—differ. Eleven out of 26 countries use the *stock*-*change approach* in their GHGIs [5], implemented either every year (using any available new data) or at the end of each NFI cycle [25]. In these cases, the degree of independence between the CBM and GHGIs is very high because the GHGIs typically do not use net increment and harvest values (i.e. the most important drivers for the sink estimated by the CBM). Furthermore, even in the 15 countries that use the *gain*-*loss approach* [5]—the approach also used by the CBM—the steps needed to obtain CO_2_ emission/removals are complex and introduce uncertainty, e.g. converting net increment minus disturbance losses (harvest, storm, fire) into the sink estimate. For example, the most recent data from NFI typically used in GHGIs (e.g. on net increment) are not always publicly available, and in several cases require interpretations and/or assumptions. Equations used by CBM to convert volume into C are totally independent from GHGIs. Harvest rates for the 26 countries used by the CBM are based on FAO statistics (which often require interpretation and/or adjustments, see [23], but the GHGIs may use either FAO or other national-level statistics. In summary, in most cases the methods to estimate emission/removals should be seen as largely independent between the CBM and GHGIs.

Few studies compared model results with European countries’ GHGIs. The main comparison may be done with [7], where two models (EFISCEN and G4 M) were applied in 24 EU countries for the period 2000 to 2008, with the discussion focused on six countries. In comparison to that study, our analyses cover a longer period (2000–2012) and 26 countries and we include the DOM and mineral soils pool dynamics and the explicit simulation of the impact of natural disturbances. Beyond these differences—which in several cases allowed CBM to obtain a better match with GHGIs—most of the conclusions from Goen et al. [7] are valid also for our study, e.g. (1) in several cases (i.e., for Germany), the estimation method used in the GHGIs (stock-change vs gain-loss) explains most of the differences observed, and (2) in the remaining cases, the differences seem to have country-specific reasons, like the amount of harvest used and the way harvest losses are treated.

In addition to the above, another essential aspect is the recalculations performed annually by the countries as part of the continuous process of improving their GHGIs. Our study therefore represents a “picture” in a rather dynamic process as future changes to GHGI may affect our conclusions. The frequency of the recalculations in the LULUCF sector is high: according to EU countries’ GHGIs submitted between 2010 and 2014 (including the time series 1990–2008 and 1990–2012, respectively), and focusing only on FL–FL, on average every year 5–6 countries out of 26 revised emissions of the previous GHGI by 10–25 % (in terms of absolute level of emissions), and another 5–6 countries revise emissions by more than 25 %. This means that every year more than a third of the countries analyzed in this study show substantial recalculations compared to their previous GHGIs, with the biggest changes usually for the more recent years. These recalculations are due to a number of reasons (e.g. new input data, addition of pools or gases, correction of previous errors, change in methods, etc.), linked to country internal processes or to recommendations provided by the UNFCCC expert review teams. The magnitude of these recalculations is consistent with the information available on uncertainties from countries’ GHGIs, which for FM in most cases fall in the range of 25–50 % [2].

Overall, given the frequency and the magnitude of the changes in GHGIs—and the associated uncertainties—for a modeler it is challenging to capture all the latest data and methods used (including possible errors) in 26 different GHGIs; an improved process to share updated information by country on an ongoing basis would certainly help. Nevertheless, the large amount of work completed by implementing the CBM in 26 countries allowed us obtain satisfactory results in most of the countries analyzed, and to understand the reasons for differences in many of the remaining cases.

## Conclusions

This study implemented a consistent methodology to estimate the GHG balance in the managed forests of 26 EU countries using the CBM to estimate the historical (2000 to 2012) net CO_2_ emissions from forest management (Sensu Kyoto, i.e. forest existing before 1990) as affected by harvest and natural disturbances (storms, fires and insects). In terms of number of countries, C pools and type of disturbances simulated, to our knowledge this is the most comprehensive study of its kind to date.

The comparison of CBM results with the data reported by the countries in their GHGIs shows a good match (both in the trend and in the level) in nine cases, a partially good match (either for the trend or the level) in an additional ten cases, and an un-satisfactory match in the remaining seven cases. A successful independent country-level validation of the CBM has also been performed.

Our study confirms that, in the short period (and excluding possible effects of climate change), the main factors driving the forest C sink of Europe’s managed forests are the harvest rates and natural disturbances (storms for most countries). When these factors are considered in a consistent way, i.e. the gain-loss method is used in both the CBM and the GHGIs, the trends of net CO_2_ emissions are very similar. Where the comparison between the CBM and the GHGIs was not fully satisfactory (for the trend and/or for the level), in most cases we provided possible explanations for the discrepancies observed, including: (1) representation of the interannual variability due to harvest and natural disturbances: while it is well simulated by the CBM, it may be masked if the country uses the stock-change approach for the GHGIs; (2) a different treatment of non-biomass pools (not reported by several countries, or reported using different assumptions compared to the CBM), or of CO_2_ emissions from fires, natural mortality or other parameters (e.g. harvest residues). Beyond these explanations, some cases—e.g. where the GHGI counter intuitively reports an increasing biomass sink associated with a trend of increasing harvest rates—clearly deserve further analysis, to identify the possible cause of the discrepancy. In general, the results of the comparisons were good in those countries where the input data for the model were based on accessible recent statistics. Finally, when analyzing the discrepancies between the CBM results and the GHGIs, it should be noted that the frequent update cycle and recalculations of GHGIs can only be reflected in the model results if national statistics on harvest and disturbance rates are readily available for the model analyses.

Overall, this study documents a promising foundation for the use of the CBM both as tool to help countries in estimating the forest C dynamics (e.g., including natural disturbances) and as a potential tool to support the verification of GHGIs at the EU level using a consistent methodological approach for all countries. A systematic comparison of the CBM with the GHGIs will certainly require additional efforts—that will require close cooperation between modelers and country experts—and caution should be applied when interpreting these first results. Nevertheless, this application of consistent methods makes a useful contribution to the continuous improvement of GHGIs, because it may help in identifying possible errors, in increasing scientific understanding and ultimately in building confidence in the estimates of emissions and removals reported by the countries by increasing consistency, transparency and completeness of the estimates.

## Methods

### The Carbon Budget Model (CBM-CFS3) and the main input data

The CBM is an inventory-based, yield-data driven model that simulates the stand- and landscape-level C dynamics of above- and below-ground biomass, dead organic matter (DOM: litter and dead wood) and mineral soil [8]. The model, developed by the Canadian Forest Service (the model description is available to the following URL: http://www.nrcan.gc.ca/forests/climate-change/carbon-accounting/13107), was recently applied to the Italian forests, in order to test the CBM for different European silvicultural systems, proposing a novel approach to include uneven-aged forest structures [13].

Because this work applies the same general assumptions used in the Italian case study, we provide only a short description of the model, highlighting the specific methodological assumptions related to the present study. Further details of the model can be found in [8], and its applications to European countries are found in [13–15].

The spatial framework applied by the CBM conceptually follows reporting method 1 ([4]) in which the spatial units are defined by their geographic boundaries and all forest stands are geographically referenced to a spatial unit (SPU). We considered 26 administrative units (i.e., European countries, as reported by Table 1) and 35 climatic units (CLUs, as defined by [26]) for a total of 910 SPUs. The CLU’s mean annual temperatures, range from −7.5 to +17.5. Each SPU was linked to a CLU through the information provided by Corine Land Cover.

**Table 1:** Summary of the main parameters applied by the CBM model for each country

Country	Original NFI year	Time step 0 (years)	CBM FM area (Mha)^a^	Harvest rate (av. 2000–2012, Mm^3^)	County specific biomass equations
Austria	2008	1998	3.2	22.9	X
Belgium	1999	1999	0.7	4.3	
Bulgaria	2000	2000	3.2	5.3	
Croatia	2006^b^	1996	2.0	4.6	
Czech Republic	2000	2000	2.6	17.0	X
Denmark	2004	1994	0.5	2.3	
Estonia	2000	2000	2.1	7.9	
Finland	1999	1999	21.7	55.0	
France	2008	1998	14.6	54.9	
Germany	2002	1992	10.6	74.7	X
Greece	1992^b^	1992	1.2	1.6	
Hungary	2008	1998	1.6	6.2	X
Ireland	2005	1995	0.5	2.8	
Italy	2005	1995	7.4	10.2	X
Latvia	2009	1999	3.2	15.8	X
Lithuania	2006	1996	2.0	7.7	
Luxembourg	1999	1999	0.1	0.3	
Netherlands	1997	1997	0.3	1.2	
Poland	1993	1993	8.9	37.8	
Portugal	2005	1995	3.6	12.2	X
Romania	1985	1985	6.6	17.2	X
Slovakia	2000	2000	1.9	9.0	
Slovenia	2000	2000	1.1	3.3	
Spain	2002	1992	12.6	16.8	
Sweden	2006	1996	22.6	79.5	
United Kingd.	1997	1997	2.5	9.8	
EU			137.9	480.7	8 countries

The table reports the NFI original reference year; the year since the model was applied; the FM area used by CBM at time step 0; the average harvest rate used; the countries where specific equations to convert the merchantable volume into aboveground biomass were selected. Two countries were not modeled: Cyprus (no NFI data available) and Malta (very small forest area, mainly covered by shrub lands)

^a^FM area used by CBM at time step 0. According to KP rules, FM is the area of forest in 1990, decreased by any subsequent deforestation. The FM area is taken from the official submissions made by countries to UNFCCC/Kyoto Protocol [18, 29], giving priority to data from KP-CRF tables when available (i.e., if FM had been elected during the first KP commitment period), or alternatively taking data from the Convention CRF tables (using ‘forest land remaining forest land’ in 1990 as a proxy for FM). To obtain FM area at time step 0, the D area reported by all countries under the Kyoto Protocol was used. Please note that CBM runs did not include forests reported as “not productive” (e.g., 0.4 Mha in Austria, 0.02 Mha in Bulgaria, 5 Mha in Sweden) and overseas territories (8.2 Mha in France)

^b^Analysis based on data from Forest Management Plans

The total managed forest area of the 26 EU countries represented here covers about 138 Mha (i.e., about 82 % of the EU forest area). Two EU countries excluded from the analysis are Cyprus (no NFI data available) and Malta (very small forest area, mainly covered by shrub lands).

Within a SPU, each forest stand is characterized by age, area and seven classifiers that provide administrative and ecological information, the link to the appropriate yield curves, and parameters defining the silvicultural system such as forest composition and management type (MT), and the main use of the harvest provided by each SPU, as fuelwood or industrial roundwood. For each country, these parameters were mainly derived from NFIs. According to country-specific information, MTs may include even-aged high forests, uneven-aged high forests, coppices and specific silvicultural systems such as clear-cuts (with different rotation lengths for each forest type, FT), thinnings, shelterwood systems, and partial cuttings.

Species-specific, stand-level equations [27] convert merchantable volume production into aboveground biomass, partitioned into merchantable stem wood, other (tops, branches, sub-merchantable size trees) and foliage components [8]. Where additional information provided by NFIs or by literature was available (see last column in Table 1), country-specific equations were selected to convert the merchantable volume into aboveground biomass [13]. If no data were available, we used the same equations selected for other countries and similar forest types (FTs, defined according to the main species). Belowground biomass is calculated using the equations provided by [28] and the annual dead wood and foliage input is estimated as a pool-specific turnover rate (percentage) applied to the standing biomass stock.

Forest inventories typically contain no or only insufficient data on stocks in DOM and soil C pools. The model therefore uses an initialization process to estimate the size of all DOM pools at the start of the simulation and then, following IPCC guidance, links DOM dynamics to biomass dynamics. Inputs from biomass to DOM pools result from biomass litterfall and turnover as well as natural and human-caused disturbances. The DOM parameters were first calibrated in the Italian cases study (see [13], Appendix E for further details), then validated on a specific study at regional level [14] and, if necessary, further modified for specific countries, such as Finland and Sweden.

We use two sets of yield tables (YT) in these analyses [13]. Historical YTs derived from the standing volumes per age class reported by the NFI represent the impacts of growth and partial disturbances during stand development. Current YTs derived from the current annual increment reported in country NFIs represent the stand-level volume accumulation in the absence of natural disturbances and management practices.

To implement the CBM to uneven-aged FTs (when this forest structure was observed in a country), all the uneven-aged forest area was allocated to a reference age class, with the average volume equal to the volume reported by the NFI for these stands. Starting from this age class, a decreasing percentage increment was applied to the subsequent (older) age classes. We assumed that, after a certain number of years, equal to species-specific cutting cycles defined at country level, each uneven-aged stand was disturbed and moved back to the initial reference age class [13]. This approach was tested through a number of simulations in which we varied different parameters. Overall, we simulated (1) a faster (but decreasing) re-growth phase during the first period following the partial cut and (2) a decreasing growth phase during the following years.

Since this study aimed to be as comparable as possible with countries’ information reported to the UNFCCC and its KP, the model was applied individually to each country and we modeled ‘forest management’ (FM) as the forests existing in 1990 minus any deforestation (D) since 1990. Forest area in 1990 and deforestation rates were obtained, respectively, from the 2014 GHGIs submitted by each country to the UNFCCC and to the KP [29]. The start year of the simulations (time step 0) varied between countries. FM area was reduced, during the model run, due to D between 1990 and time step 0. The D area within each country was distributed proportionally to the area of each FT. Table 1 shows the country-specific FM area at the start of model runs.

In order to provide a comparable dataset for all the EU countries, covering the period 2000–2012, when the NFI reference year was after the year 2000 (see Table 1), the original NFI age-class distribution (for even-aged forests) was rolled back by 10 years (see [13] for further details).

### Harvest rate

To provide a consistent estimate of the harvest demand for all 26 EU countries, historical data on harvest were obtained from FAO statistics [30]. For some countries, the original FAOSTAT data were slightly modified to ensure consistency with other information provided by countries under the KP. The country-specific modifications applied to the original FAOSTAT data (in most cases due to different treatment of the bark fraction) are described in [23].

FAOSTAT data (modified where necessary) were further distinguished at the country level, between four compartments: Industrial Roundwood (IRW, i.e., the portion of roundwood used for the production of wood commodities) and Fuelwood (FW, i.e., wood for energy use) and between coniferous and non-coniferous (i.e., for our analysis, broadleaved) species groups [30]. For each compartment, we defined in CBM: (1) the FTs (i.e., broadleaved species for IRW and FW broadleaved species, and coniferous species for IRW and FW coniferous species), (2) the MTs (for example coppices for FW from broadleaved species) and (3) the silvicultural practices (for example thinnings for FW from coniferous species) providing the total amount of wood expected each year (the harvest target).

We assumed that the harvest rate was entirely satisfied by the FM area, considering that the possible amount of harvest provided by lands afforested or reforested (AR) since 1990 was generally negligible [15], with the exception of Portugal (see the Additional file: 1 for details).

### Natural disturbances

For each country, the historical effects of storms and ice (15 countries), fires (11 countries) and insect attacks (i.e., bark beetles attacks, for 2 countries) were analysed (see Table 2 for details). We assumed that that natural disturbances occurred on the FM area, excluding possible disturbances on the afforested area.

**Table 2:** Overview of countries with natural disturbance events simulated by the CBM (*F* fire, *S* storms and ice sleets, *I* insect attacks), with information on input data used for storms (country data, National Inventory Reports, NIR or the FORESTORMS database [31] and the average annual burned area

Country	Natural disturb.	Storms, ice and insect disturbances		Fires
		Source	Vol. affected^a^ (Mm^3^ year^−1^)	Area burned^b^ (kha year^−1^)
Austria	S + I	Vol. based on country data	4.1	–
Belgium	–			–
Bulgaria	–			–
Croatia	F			2.3
Czech Rep.	F			0.5
Denmark	S	Vol. based on country data	0.5	–
Estonia	S	Area and vol. based on NIR	0.7	–
Finland	S	Vol. based on FORESTORMS	0.6	–
France	S	Area and vol. based on FORESTORMS	18.3	–
Germany	S	Vol. based on FORESTORMS	6.2	–
Greece	F			6.0
Hungary	–			–
Ireland	F			0.4
Italy	F			35.0
Latvia	S	Vol. based on FORESTORMS	0.7	–
Lithuania	S + F + I	Vol. based on the NIR + FORESTORMS	0.2	0.3
Luxembourg	S	Vol. based on FORESTORMS	<0.1	–
Netherlands	S	Vol. based on FORESTORMS	<0.1	–
Poland	S	Vol. based on FORESTORMS	0.4	–
Portugal	F			49.1
Romania	–			–
Slovakia	S + F	Vol. based on FORESTORMS + country data	0.8	0.6
Slovenia	S + F	Vol. based on country data	<0.1	0.1
Spain	F			35.3
Sweden	S	Vol. based on FORESTORMS + country data	7.1	–
United K.	S + F	Vol. based on FORESTORMS	<0.1	3.5
	22 countries		39.6*	134.0

^a^Average volume affected by storms, ice and insects between 2000–2012, as reported by the input data used by CBM. The interannual variations of these disturbances can vary considerably among countries (i.e., in many cases disturbances are concentrated in few big events). In some cases, further damages were considered before 2000

^b^Average area affected by fires between 2000–2012, mainly based on the data reported by National Inventory Reports*

The effect of storms was evaluated using the data reported by the FORESTORMS database [31] provided by the European Forest Institute and by specific additional information available at the country level. Depending on the available information, the effect of each event was modelled according to (1) the amount of forest biomass damaged by storm and eventually salvage logged and/or (2) the amount of area affected by the disturbance event. In the first case, we mainly modified the ‘disturbance matrix’ that describes the proportion of C transferred between pools and to the forest product sector or released to the atmosphere [8], in order to be consistent with the disturbance impact reported by the FORESTORMS database. In the second case, we verified that the amount of forest area affected by the disturbance event was consistent with the area reported by this database. In some cases, such as for Sweden, both these criteria were verified.

More specific information on the methodological assumptions applied to represent storms and insect attacks are reported in the Additional file: 1 for some representative case study. Since the information available on these disturbances may vary considerably by country, our assumptions were adapted to the conditions in each country.

Fire disturbances were modelled according to the amount of area affected by fire, as reported by national statistics, proportionally distributed between different FTs or according to further information provided by literature (mainly, the National Inventory Reports) The disturbance matrix associated with fires was modified according to specific country-level information, to account for salvage of logging residues, commonly applied in some Mediterranean countries (i.e., Portugal). More specific information on the methodological assumptions applied to these disturbances is reported in the Additional file:1 for Portugal. As in the case of storms, our model assumptions were adapted to the specific country’s conditions. When relevant (e.g., for Latvia), we also included the burning of harvest residues after a clearcut.

### Model validation

For Lithuania, the information provided by CBM, based on Lithuania’s NFI used as input data for the model, can be also compared and validated against some independent data, derived by specific studies^4^ on living and dead tree volumes in forest land, reported by Lithuania’s NIR [17]. Further details on the methodological assumptions are reported in the Additional file: 1.

## Additional file

**Additional file 1.** Country case studies: Austria, Germany, Lithuania, Poland, Portugal

## Abbreviations

AR: afforestation and reforestation
C: carbon
CBM: Carbon Budget Model
CP1: first commitment period
CP2: second commitment period
D: deforestation
DOM: dead organic matter
EU: European Union
FL: forest land
FM: forest management
FRA: forest resources assessment
FT: forest type
FW: fuelwood
GHG: greenhouse gas
GHGI: greenhouse gas inventory
HWP: harvested wood product
IPCC: Intergovernmental Panel on Climate Change
IRW: industrial roundwood
KP: Kyoto protocol
LULUCF: land use, land-use change and forestry
MT: management type
NFIs: National Forest Inventories
NIR: National Inventory Report
SPU: spatial unit
UNFCCC: United Nations Framework Convention on Climate Change
YT: yield table

## Footnotes

1 i.e. Transparency, accuracy, completeness, consistency, comparability.

2 When available, FM country data from the KP-CRF tables was used for 2008-2012 (i.e., if FM had been elected during the first KP commitment period); alternatively, country data were taken from the Convention CRF tables using ‘forest land remaining forest land’ (FL remaining FL) as a proxy for FM.

3 This value is in the lower part of the range of uncertainties typically reported by EU countries for FM emissions/removals (25-50 %, EU NIR 2014).

4 “Study 1, “Forest Land Changes in Lithuania during 1990–2001” ([17], page. 349).
