# Supplementary material for: Modelling forest carbon stock changes as affected by harvest and natural disturbances. I. Comparison with countries’ estimates for forest management
Source: Carbon Balance Manag. 2016 May 23;11:5. doi: 10.1186/s13021-016-0047-8 (PMC4877427; doi:10.1186/s13021-016-0047-8)
Supplement: Supplementary file 1 — Additional file 1. Country case studies: Austria, Germany, Lithuania, Poland, Portugal [file 13021_2016_47_MOESM1_ESM.docx]

# Supplementary materials

## Austria

### Methodological assumptions

The analysis was based on the data provided by NFI 2007-2009 ([1]). Assuming 2008 as NFI reference year, all data were brought back to 1998. The total forest area was distributed between 9 administrative regions and 12 Climatic units.

According to the information provided by NFI, the following management compartments were considered:

1. Productive forests
2. Protective and productive forests
3. Coppices

TOTAL Productive forests 3,380 kha

1. Protective and other “not productive” forests 597 kha

TOTAL 3,977 kha

For (i) and (ii): the age class distribution by species and regions was downloaded by the NFI web site.

For (iii): the age total class distribution by regions was downloaded by the NFI web site.

For (iv): the age class, species and regional distribution was argued according to the proportion of area assigned to (i)

All age classes were made uniform to 10 years span.

According to the 2014 CRF Tables ([2] Tab. 5.a), the 1990 FL remaining FL area was equal to 3,224 kha (productive forests), plus the forests out of yield, equal to 407 kha. Based on the 2014 NIR ([3], pag. 354), “*the calculations of C-losses and C-gains for FL remaining FL consider only the area of* ***productive forest (forest in yield)****. The assumption for the exclusion of carbon stock changes in nonproductive forests is the following: There is a balance between C-losses due to decay and C gains due to biomass increment. There is no extraction of biomass due to the missing access to these forests, but the opposite, planting measures are carried out (for maintaining the essential protective function of these forests)*”.

We scaled the total “Productive Forest area” to 3,224 kha, assumed as FM area, according to the following assumptions:

1. The difference between the Productive Forest area reported by NFI and the FM area, equal to 156,915 ha, was assumed as new forest area (AR), established between 1990 and 2008 (i.e., 18 years).
2. About 55% (i.e., 10/18) of this forest expansion was assigned to the first age class (≤ 10 yrs.), referred to new forest stands less than 10 years old. The area of the first age class was therefore reduced of 87,175 ha.
3. The remaining fraction (i.e., 8/18) was assigned to the second age class (between 11 and 20 yrs.) and it is referred to new forest stands older than 10 years. The area of the first age classes was therefore reduced of 69,740 ha.
4. Each even-aged high forest stand (excluding any expansion on coppices) belonging to the first and the second age class was proportionally reduced according to the previous assumptions.
5. The final area was further decreased to about 3,198 kha, to account for the amount of deforestation occurred between 1990 and 1998 (equal to 3,260 ha yr^-1^).

Protective and other “not productive forests” were not considered by the present analysis.

Species-specific, stand-level equations ([4]) were selected to convert merchantable volume production into aboveground biomass ([5]), using additional information provided by the Austrian NFIs and by literature ([6]). In particular, we considered the total aboveground volume reported by NFI and we estimated the biomass through the following assumptions:

- 1. Volume per ha (m^3^ ha^-1^): Vol_ha_ = Tot Volume/ forest area
  2. Wood density (WD, t dry matter m^-3^): 0.39 for conifers 0.53 for broadleaves
  3. BEFs (default IPCC values): 1.3 for conifers 1.4 for broadleaves

The total aboveground biomass (*Tot AG*) was estimated as:

Tot AG =Vol_ha_ * WD * BEFs Eq. (1)

To estimate the aboveground biomass from the volume-based yield tables, the CBM applies to each single species (or, as in this case, forest type) stand-level equations (5]) developed by [4]. Each of the main Austrian forest types (FTs) were associated to an appropriate Canadian species (Tab. 1) following the approach described in [6].

| **Forest type** | **Acronym** | **Species selected by default CBM database (Boudewyn et al., 2007)** | **Mean Δ** | **St dev.** |
| --- | --- | --- | --- | --- |
| Spruce | PA | Eastern white cedar (Thuja occid.) | 1.87 | 6.95 |
| Fir | AA | Eastern white pine (P. strobus) | -0.66 | 3.17 |
| Larch | LD | Red pine (P. resinosa) | 2.35 | 2.11 |
| Black pine | PN | Eastern white cedar (Thuja occid.) | - | - |
| Other conifers | OC | Eastern white cedar (Thuja occid.) | - | - |
| Beech | FS | White ash (Fraxinus americana) | 2.88 | 2.54 |
| Oaks | QR | Black cherry (Prunus serotina) | 9.02 | 4.00 |
| Other hardwoods | OH | Largetooth aspen (Populus grandidentata) | 1.63 | 3.89 |
| Other broadl. | OB | Basswood (Tilia americana) | 3.65 | 6.59 |

**Tab. 1: the table reports: (i) the main forest types (FTs) defined for Austria; (ii) the acronym applied to each FT; (iii) the original species from which were derived the equations selected for each forest type, according to the methodological assumptions reported by [6]; (iv) the mean percentage difference and (v) the standard deviation between the average aboveground total biomass estimated by the selected equations and the reference country-specific biomass values. For PN and OC, due to the low number of observations, the same equations selected for PA were applied.**

Species-specific YTs were selected using the average volume and increment reported by NFI.

The original increment values reported by NFI are referred to the increase of tree stem volume over bark between two inventories, excluding harvested trees and natural trees that have died from natural causes ([7]). In order to estimate the net growth including the amount of harvested trees, the following equations were applied:

$Pi=\frac{I_{NFI}}{V_{NFI}}$ Eq. (2)

$CAI={(V}_{NFI}+D)\times Pi$ Eq. (3)

where, *P_i_* is the percentage increment estimated on the original net increment (*I_NFI_*) and volume (*V_NFI_*) reported by NFI; *CAI* is the current net annual increment and *D* (drains) consists of the stem volumes of cut trees and trees that have disappeared from the collective of sample trees due to mortality or natural causes. The total amount of drains (by regions and species) reported by the Austrian NFI, was divided by the total amount of productive forest area, in order to estimate the amount of drains by hectares and species.

Tab. 2 reports the main species grouped by FTs and the minimum rotation length applied to clear cuts. About 45% the harvest demand was also provided by thinnings (removing between 15 – 30% of the merchantable biomass, depending by FTs and Management Types, MTs).

| **CBM**  **Forest Types** | **Manag. Type** | **Minimum rotation length (yrs)** | **Species reported by NFI** |
| --- | --- | --- | --- |
| PA | H | 110 - 120 | Fichte (P. abies) |
| AA | H | 110 | Tanne (A. alba) |
| LD | H | 120 | Larch (L. decidua) |
| PN | H | 80 - 100 | Schwarzkiefer (P. nigra) |
| OC | H | 80 | Sonstiges Nadelholz (other conifers) |
| FS | H | 110 - 120 | Buche (F. sylvatica) |
| QR | H | 80 | Eiche (Quercus sp.) |
| OS | H | 30 - 40 | Weichlaub(Other broadl. i.e Betula, Populus, Alnus) |
| OH | H | 70 | Sonstiges Hartlaub (hardwood broadl. i.e Fraxinus, Acer) |
| OH | C | 30 | Broadleaves hardwoods |
| PS | H | 80 - 90 | Weisskiefer (P. sabiniana) |
| PC | H | 110 - 120 | Zirbe (P. cembra) |

Tab. 2: the table reports the forest types applied by CBM, the corresponding species reported by NFI and the minimum rotation length applied to clear cuts for the Productive high forests (H) and coppices (C) management types.

We considered the effect of natural disturbance events due to storms, snow and insects attacks affecting the Austrian forests. The amount of merchantable volume damaged by storms and snow between 1998 and 2007 (reported in Tab. 3) was derived by the 2008 Austrian Forest Report ([9]). The amount of merchantable volume damaged between 2008 and 2012 was estimated as the average of the previous period.

| **Step** | **Year** | **Vol. damaged by Storm and snow** | **Vol. damaged by**  **Bark beetle** |
| --- | --- | --- | --- |
| 1 | 1998 | 1.2 | 0.8 |
| 2 | 1999 | 1.3 | 0.7 |
| 3 | 2000 | 1.2 | 0.6 |
| 4 | 2001 | 0.4 | 0.7 |
| 5 | 2002 | 6.1 | 0.8 |
| 6 | 2003 | 1.2 | 2.0 |
| 7 | 2004 | 1.1 | 2.4 |
| 8 | 2005 | 0.8 | 2.5 |
| 9 | 2006 | 2.8 | 2.4 |
| 10 | 2007 | 5.0 | 2.3* |
| 11 | 2008^+^ | 2.1^+^ | 1.4^^^ |
| 12 | 2009 | 2.1^+^ | 1.4^^^ |
| 13 | 2010 | 2.1^+^ | 1.4^^^ |
| 14 | 2011 | 2.1^+^ | 1.4^^^ |
| 15 | 2012 | 2.1^+^ | 1.4^^^ |
| * Average 2003 - 2006 | | | |
| ^+^ Average 1998 - 2007 | | | |
| ^^^ Average 1998 - 2006 | | | |

Tab. 3: merchantable volume (M m^3^) damaged by storms and snow between 1998 and 2008 and by bark beetle between 1998 and 2007 (all values were derived by the figures reported on pg. 23 of the 2008 Austrian Forest Report, [9]). From 2007 to 2012 the average amount of volume damaged in the previous period was estimated and applied during the model run.

The effect of storms was modelled through four different disturbance events: (i) a stand replacing-storm (and snow) disturbance event; (ii) a widespread-storm (and snow) disturbance; (iii) a specific disturbance event for modelling the bark beetle attack and (iv) a disturbance event for modelling salvage of logging residues after widespread-storms and bark beetle attacks. The analysis was based on the following assumptions:

1. For the stand-replacing storm and snow disturbance event:
2. We assumed that a fraction of the forest area reported by the first age class (i.e., < 10 yrs., assumed as clear-cut forest area) in the original age class distribution (i.e., referred to 2008) was affected by stand replacing-storm between 1998 and 2008.
3. Comparing the annual amount of damaged volume (*Vol_St_*) with each harvest compartment, as derived by FAO statistics (IRW^[[1]](#footnote-1)^ conifers, IRW Broadleaves, FW^[[2]](#footnote-2)^ conifers and FW broadleaves) we highlighted that the amount of damaged harvested trees was only related to the IRW conifers (*IRW*_*C*) compartment.
4. We estimated the share of *IRW*_C potentially provided by trees damaged by storms and snow, named Storm Factor (*SF*) and equal to:

$SF=\frac{{Vol}_{St}}{IRW\_C}$ Eq. (4)

1. We assumed that the amount of forest area affected by stand replacing-storm, each year, was equal to the clear-cut forest area of the *i-*year multiplied by the Storm Factor.
2. We assumed that this disturbance only affected spruce forests (i.e., the main coniferous species in Austria) with an average age between 70 and 80 years (i.e., the average age of the spruce Austrian forests according to NFI data).
3. Storms and snows disturbances were simulated through a clear-cut, stand replacing, disturbance event, assuming that 95% of the merchantable living biomass was moved to the products pool (i.e., harvested after the disturbance event and therefore not accounted under DOM pool) and the other merchantable living biomass components were moved to dead wood and litter pools.
4. For the widespread storm and snow disturbance event:
   1. After a preliminary run, based on the assumption reported above, we estimated the amount of merchantable biomass (in tons of C) damaged by stand-replacing storm (V_St_St_Repl_).
   2. The biomass affected by widespread disturbance events (V_St_St_W_) was estimated as the difference between the total merchantable biomass (expressed as tons of C) damaged by storm (see Tab. 4) and V_St_St_Repl_.
   3. Assuming that: (i) even this event was mainly affecting spruce; (ii) it was affecting 40% of the merchantable biomass pool and (iii) considering the area affected by the stand-replacing disturbance (provided by preliminary runs), we estimated the amount of area requested, in order to satisfy the expected amount of biomass damaged (i.e., V_St_St_W_).

| **Year** | Total Storm & snow damage | | Stand Repl. Dist. | Widespread Dist. | |
| --- | --- | --- | --- | --- | --- |
|  | **Tot_Vol**  **(M m^3^)** | B= (Tot_Vol/2)*0.40*10^6^  (tons C) | C=CBM Output  (tons C) | **D=B-C**  (tons C) | **Area* (ha)** |
| 1998 | 1.2 | 240,000 | 151,353 | 88,647 | 3,911 |
| 1999 | 1.3 | 260,000 | 162,810 | 97,190 | 4,214 |
| 2000 | 1.2 | 240,000 | 152,314 | 87,686 | 3,886 |
| 2001 | 0.4 | 80,000 | 51,204 | 28,796 | 1,300 |
| 2002 | 6.1 | 1,220,000 | 758,154 | 461,846 | 19,485 |
| 2003 | 1.2 | 240,000 | 144,103 | 95,897 | 3,757 |
| 2004 | 1.1 | 220,000 | 127,419 | 92,581 | 3,553 |
| 2005 | 0.8 | 160,000 | 95,818 | 64,182 | 2,608 |
| 2006 | 2.8 | 560,000 | 325,822 | 234,178 | 9,293 |
| 2007 | 5.0 | 1,000,000 | 533,900 | 466,100 | 15,917 |
| 2008^+^ | 2.1 | 422,000 | 224,420 | 171,710 | 6,792 |
| 2009 | 2.1 | 422,000 | 216,626 | 171,710 | 6,792 |
| 2010 | 2.1 | 422,000 | 222,452 | 171,710 | 6,792 |
| 2011 | 2.1 | 422,000 | 222,414 | 171,710 | 6,792 |
| 2012 | 2.1 | 422,000 | 229,696 | 171,710 | 6,792 |
| * The final area was estimated after some preliminary simulation, in order to satisfy the expected amount of biomass damaged (column D). | | | | | |

Tab. 4: the table summarizes the assumptions made to distribute the total amount of biomass damaged by storm and snow disturbances, distributed between stand-replacing disturbance events and widespread disturbances. In this last case, after a preliminary run we estimated the amount of biomass (in tons of C) affected by stand-replacing disturbance events (column C). The difference between this amount and the total amount of biomass damaged by storm (column B) is equal to the amount of biomass damaged by widespread disturbances (column D); the area was estimated through some further preliminary runs as the amount of area providing the values expected in column D.

- 1. We assumed that there was no direct salvage of logging residues associated with the widespread storm, i.e., 40% of the living biomass was moved to DOM pool.

1. For the bark beetle attacks on spruce forests:
   1. The merchantable volume damaged (reported in Tab. 3) was converted to tons of C (assuming an average wood density equal to 0.4 for spruce) and divided by the average merchantable spruce biomass per ha estimated through a preliminary run. This value (in t C ha^-1^) was finally multiplied for a constant factor equal to 0.05, assuming that 5% of the living biomass was affected by this disturbance event (see Tab. 5).

| Year | Vol damaged (M m3) | t C PA | Avg Merch Biom PA (t C ha-1) | Area (ha) | Area Assuming 5% damage (ha) |
| --- | --- | --- | --- | --- | --- |
| COLUMN | **A** | **B=**A*10^6^*0.5*0.4 | **C** | **D=**B/C | **E=**B/(C*0.05) |
| 1997 | 1 | 200,000 | 48 | 4,169 | 83,386 |
| 1998 | 0.8 | 160,000 | 49 | 3,254 | 65,089 |
| 1999 | 0.7 | 140,000 | 50 | 2,780 | 55,597 |
| 2000 | 0.6 | 120,000 | 52 | 2,324 | 46,488 |
| 2001 | 0.7 | 140,000 | 53 | 2,648 | 52,970 |
| 2002 | 0.8 | 160,000 | 54 | 2,955 | 59,093 |
| 2003 | 2 | 400,000 | 55 | 7,265 | 145,310 |
| 2004 | 2.4 | 480,000 | 56 | 8,580 | 171,608 |
| 2005 | 2.5 | 500,000 | 57 | 8,799 | 175,975 |
| 2006 | 2.4 | 480,000 | 58 | 8,331 | 166,612 |
| 2007 | 2.3 | 465,000 | 58 | 7,964 | 159,272 |
| 2008 | 1.4 | 286,667 | 58 | 4,909 | 98,189 |
| 2009 | 1.4 | 286,667 | 58 | 4,909 | 98,189 |
| 2010 | 1.4 | 286,667 | 58 | 4,909 | 98,189 |
| 2011 | 1.4 | 286,667 | 58 | 4,909 | 98,189 |
| 2012 | 1.4 | 286,667 | 58 | 4,909 | 98,189 |

Tab. 5: the table summarizes the assumptions made to estimate the spruce (PA) area affected by bark beetles attacks: (i) the total volume (column A) was converted to tons of C (column B, assuming a wood density equal to 0.4 for spruce); dividing this amount by the average merchantable biomass per ha (referred to spruce and provided by a preliminary run) we estimated the total area potentially affected by this disturbance (column D); reducing by 0.05 the average merchantable biomass per ha (i.e., assuming that bark beetles attack 5% of the living biomass), we estimated the final area affected by this disturbance (column E)

- 1. We assumed that there was no direct salvage of logging residues associated with this event, i.e., 5% of the living biomass was moved to DOM pool.

1. Salvage of logging residues after the widespread storms and insect attacks, was simulated through specific events, moving the biomass from the stem snags pools to the product pool. We assumed that salvage of logging residues was done on the same amount of area and age classes affected by these events, starting from the year after the disturbance. We also prioritized the removals of harvest residues, from the stands having the highest stem snag carbon amount^[[3]](#footnote-3)^.

### Supplementary results

The total volume damaged by natural disturbances estimated by our model is reported in Figure 1. This is consistent with our input data. Of course, since we do not have specific values on the salvage of logging residues, our assumptions on the total volume removed as harvest after the disturbance events (even reported in Figure 1) may differ from country’s data.


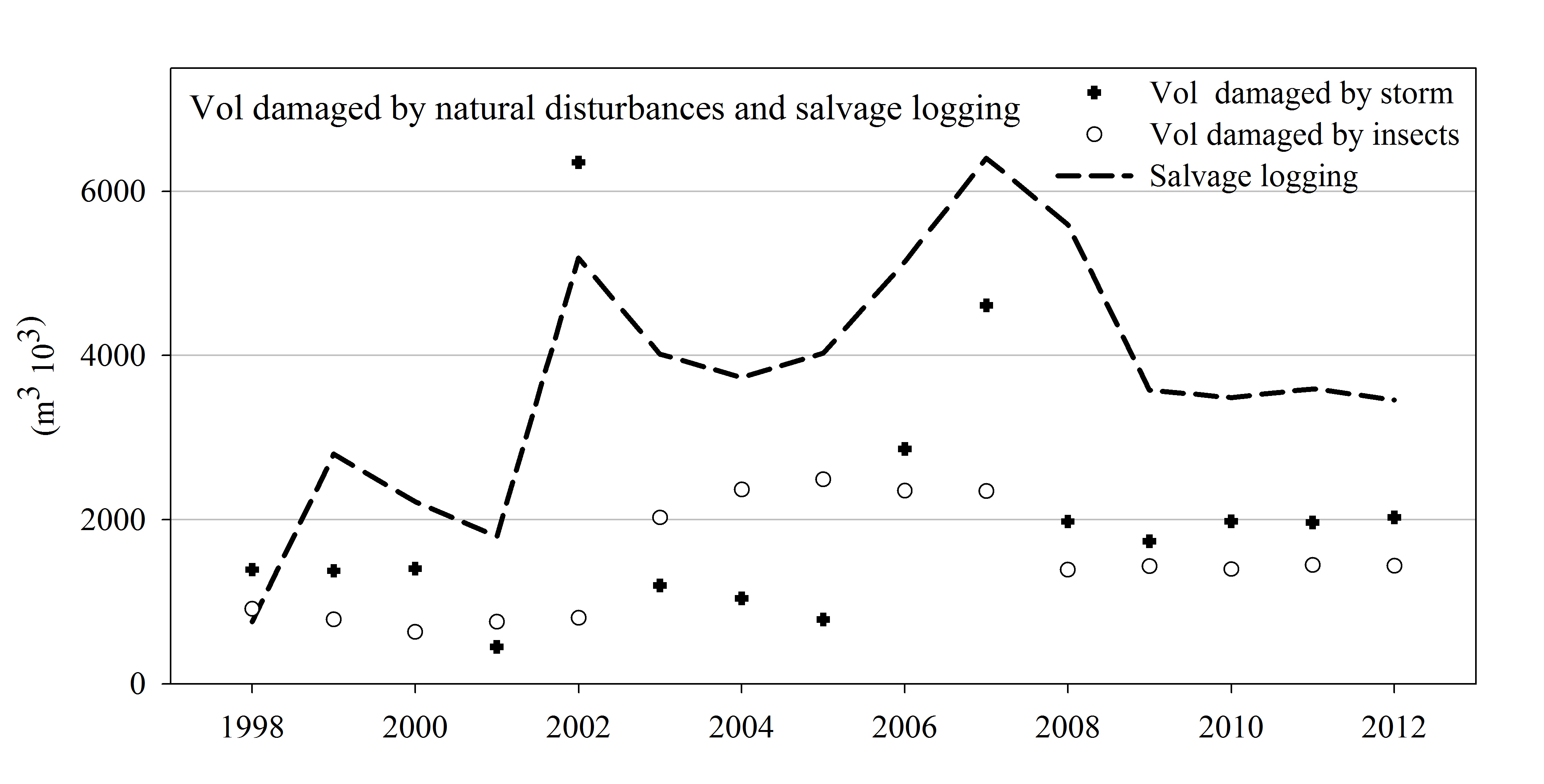
**Figure 1: merchantable volume damaged by natural disturbances (in m^3^ 10^3^), based on CBM’s output, further distinguished between the volume damaged by storms and bark beetles and the total volume of damaged trees removed as salvage of logging residues.**

Figure 2 reports the age class evolution provided by CBM model, compared with the original age class distribution reported by NFI for 2008.


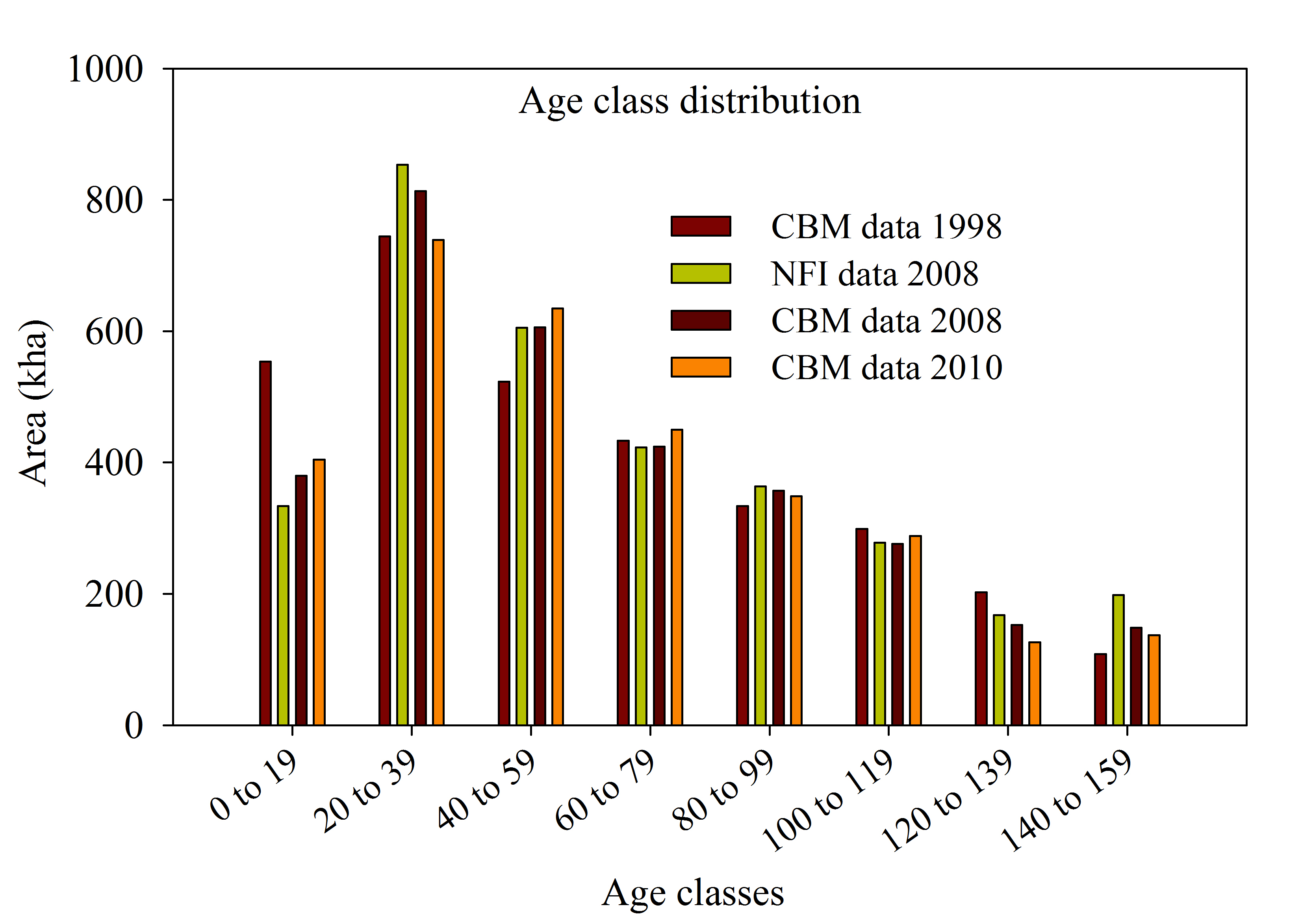


**Figure 2: age class evolution provided by CBM model between 1998 and 2012, including the effect of deforestation; the original NFI age class distribution (assigned to 2008) is also reported.**

## Germany

### Methodological assumptions

The analysis was based on the data provided by the Second German NFI, referred to 2002 ([10]). The total area reported by NFI, equal to 10,320 kha, was scaled to the 1990 FM area reported by Germany (i.e. 10,651 kha). The original data were brought back to 1992 and the total amount of FM area was further decreased to about 10,629 kha, assuming an annual rate of deforestation equal to 11,170 ha yr^-1^, applied from 1990 to 1992. All age classes were made uniform to 10 years.

Species-specific, stand-level equations ([4]) were selected to convert merchantable volume production into aboveground biomass, using additional information reported by literature. In particular, the equations provided [11]) and [12]) were applied to the average volume reported by NFI, in order to estimate the total aboveground biomass stock for each FT and to select (from the original CBM database) the equations that minimize the difference with the figures derived by the NFI ([6]). Tab. 6 reports the original species from which were derived the equations selected for each forest type.

| **Forest type** | **Acronym** | **Species selected by default CBM database** | **Minimum Rotation length (yrs)** |
| --- | --- | --- | --- |
| **Fir** | **AA** | Pinus banksianae | **100** |
| **Beech** | **FS** | Acer saccharum | **140** |
| **Larch** | **LD** | Larix laricina | **100** |
| **Other deciduous short life** | **OB_S** | Salix nigra | **40** |
| **Other deciduous long life** | **OB_L** | Acer negundo | **80** |
| **Spruce** | **PA** | Picea abies | **80** |
| **Pseudotsuga menziesii** | **PsM** | Pinus resinosa | **100** |
| **Scots pine** | **PS** | Pinus contorta | **130** |
| **Oak** | **QR** | Ostria virginiana | **150** |

Tab. 6: the table reports: (i) the main forest types (FTs) defined for Germany; (ii) the acronym applied to each FT; (iii) the original species from which were derived the equations selected for each FT, according to the methodological assumptions reported by [6]; (iv) the clearcut rotation length applied to each FT.

All forests were reported as even-aged high-forests and assumed as pure forests. The silvicultural system was based on clear-cut and thinnings. The minimum rotation length applied to clearcut (reported in Tab. 6) was defined according to additional information reported by the NIR ([13]).

The main parameters defining the harvest criteria applied by CBM for Germany are reported on Tab. 7.

| **Silvicultural treatment** | **Criteria** | **Harvest share** |
| --- | --- | --- |
| 20% Commercial Thinnings | 15 – 120 yrs | 7% |
| 30% Commercial Thinnings | 30 - 150 yrs. | 9% |
| 35% Commercial Thinnings* | > 45 yrs. | 38% |
| Clearcut (95% commercial thinning) | Depending by species | 39% |
| Salvage logging after storm events | Depending by specific events | ≈ 4% |
| * Clearcut applied to small areas, only applied to PA | | |

Tab. 7: main parameters defining the harvest criteria applied by CBM for Germany, including the age classes affected by each silvicultural treatment and the relative share of harvest provided by each treatment (average for the historical period).

Species-specific YTs were selected using the average volume and increment reported by NFI. Figure 3 reports a comparison between the CAI (in mc ha^-1^ yr^-1^) applied by CBM for the current YTs and the CAI reported by NFI. These last values were corrected to account for the amount of young plants that do not reach the minimum *Dbh* threshold (7 cm) during one year ([8]).


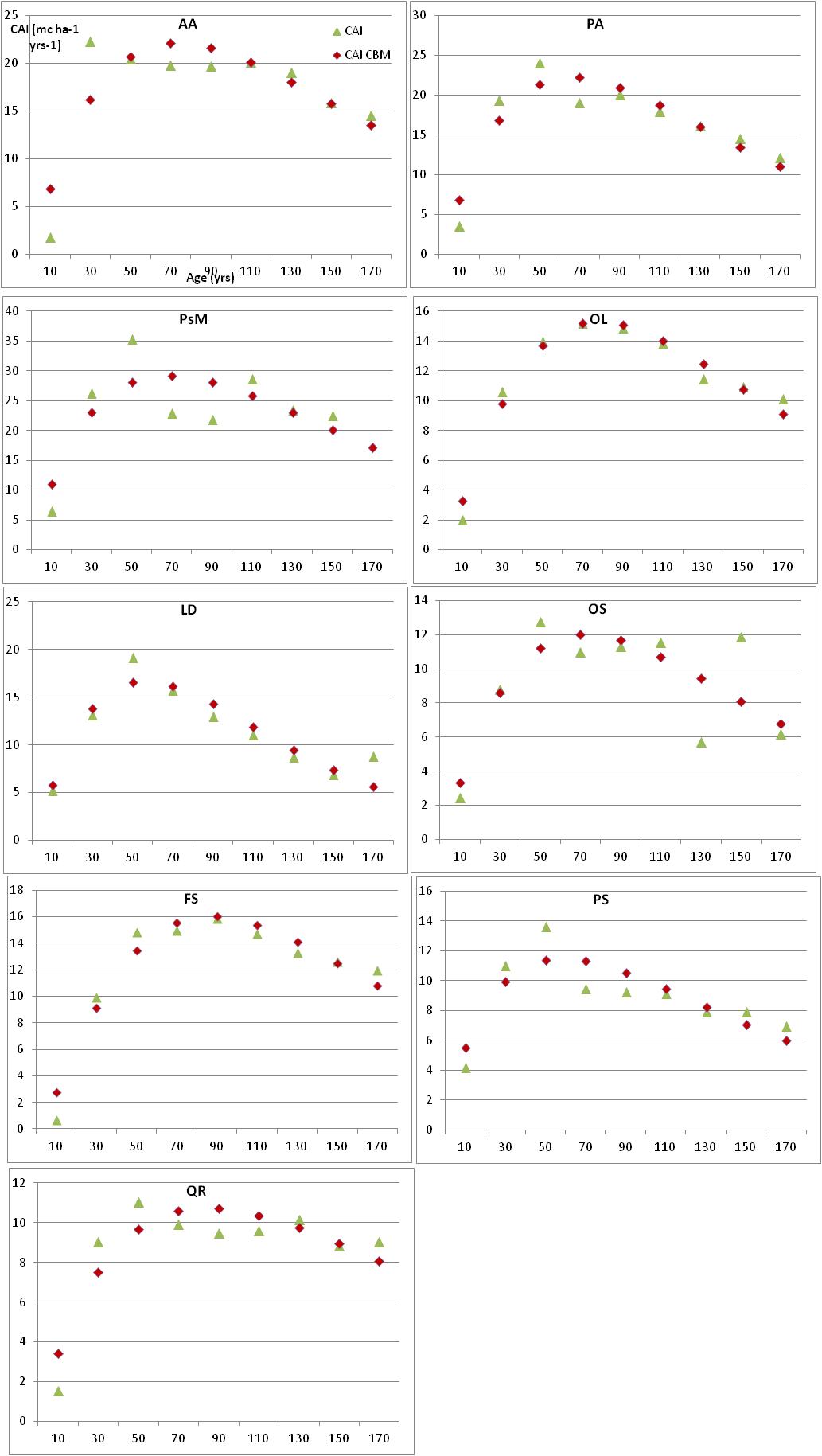


Figure 3: comparison between the CAI (in mc ha^-1^ yr^-1^) applied by CBM model for the current YTs and the CAI reported by NFI for Germany.

The effects of the main disturbance events due to wind storms were included in the model run (Tab. 8).

| **Year** | **Volume**  **damaged** |
| --- | --- |
| 1992 | - |
| 1993 | - |
| 1994 | 2,680,000 |
| 1995 | 1,330,000 |
| 1996 | - |
| 1997 | 900,000 |
| 1998 | - |
| 1999 | 34,290,000* |
| 2000 | - |
| 2001 | - |
| 2002 | - |
| 2003 | - |
| 2004 | - |
| 2005 | - |
| 2006 | - |
| 2007 | 37,000,000 |
| 2008 | - |
| 2009 | - |
| 2010 | 3,590,000 |
| * The effect of this storm, occurred on December 1999 was postponed to 2000 | |

Tab. 8: volume damaged by storms, according to the information reported by [14].

Comparing the amount of primary damaged volume reported by the FORESTORMS database ([14]) with the total harvest amount per year, we applied the following assumptions (see also Figure 4):

- 1. Considering that the storms in 1999 and 2007 had a clearly identified effect on the total amount of harvest (HWP) referred to conifers industrial roundwood (at year T=2000 for the storm occurred in 1999 and T=2007 for the storm occurred in 2007), we estimated the average amount of harvest excluding salvage logging in year T (${Hwp}_{T}^{*}$), as:

${Hwp}_{T}^{*}={HWP}_{T-1}-{HWP}_{T+1}$ Eq. (5)

- 1. The difference between *HWP_T_* (i.e. the original harvest demand) and ${Hwp}_{T}^{*}$, estimated for the disturbance events of 2000 and 2007 (for the other storms no effect on the total amount of harvest can be highlighted) represents the amount of salvage logging, i.e., about 17.6 Mm^3^ and 14.8 Mm^3^, removed in 2000 and 2007, respectively.
  2. From these two events, we estimated the average percentage amount of salvage logging, equal to 46% of the volume damaged and we applied this percentage to the other disturbance events.


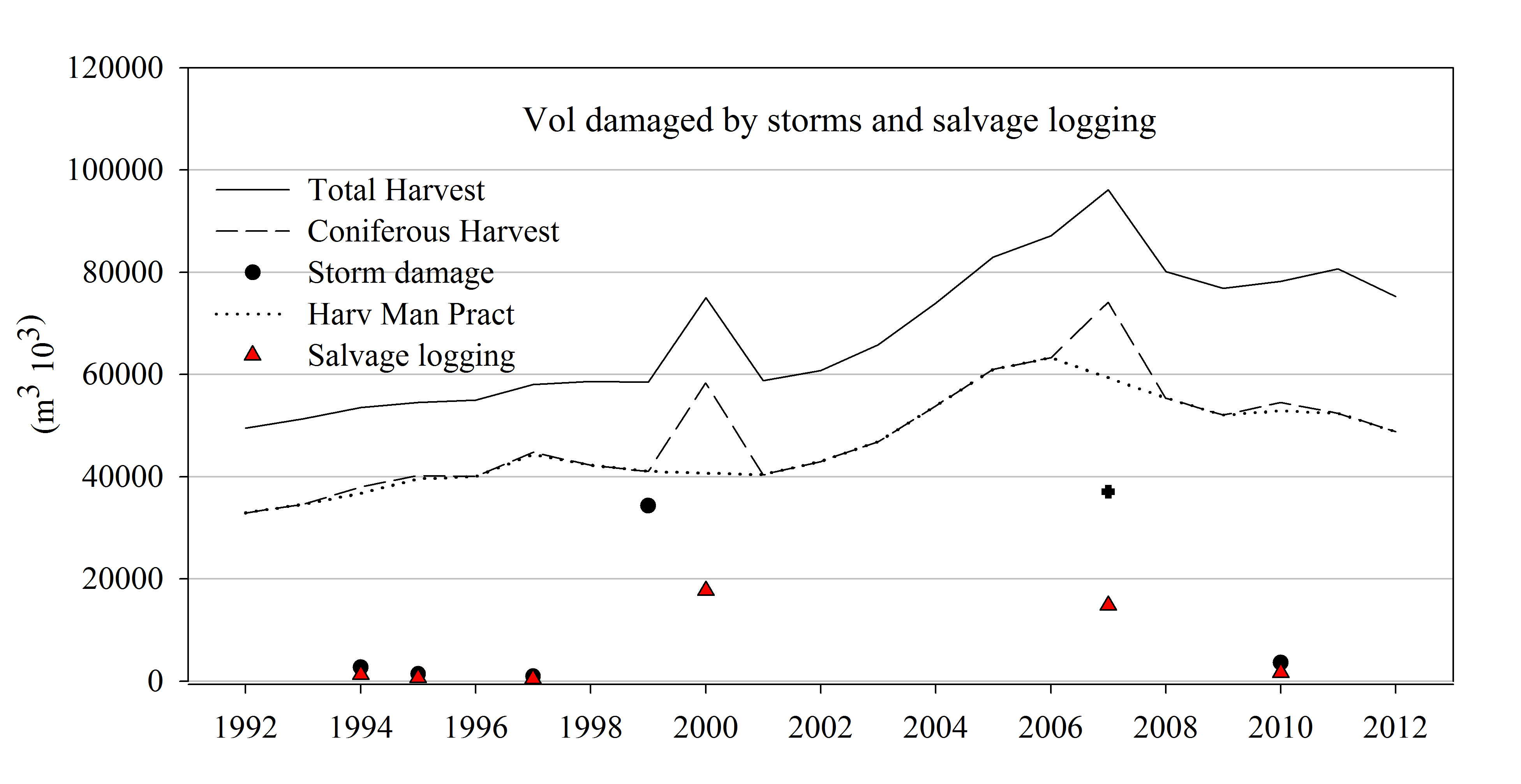


Figure 4: total harvest demand (in m^3^ 10^3^) applied to Germany (Total Harvest) between 1992 and 2012, further distinguished between: the total amount of harvest provided by conifers (Coniferous Harvest), the amount of harvest damaged by storms (Storm damage, as reported in Tab. 8), the volume removed as salvage of logging residues (Salvage logging) and the volume provided by usual management practices (Harv Man Pract).

The effect of each storm was modelled through three different disturbance events applied during the model run: (i) a stand replacing-storm disturbance event; (ii) a widespread-storm disturbance event and (iii) a specific disturbance event for salvage of logging residues during the 3 years following the storm.

1. In the stand replacing-storm disturbance event, we assumed that on average 46% of the aboveground merchantable biomass moved to the HWP pool (this is a “direct salvage logging”); the remaining fraction moves to DOM pool. This disturbance has a direct effect on the age class distribution and was modelled taking into account the evolution of the age class distribution in the first 10 years, i.e., according to the total area reported in the first age class of the original age class distribution for spruce. We assumed that this event affected spruce forests between 70 and 80 yrs old (i.e., the average age of spruce forests in 2002). This assumption allowed to reconstruct the age class distribution expected in 2002, starting from 1992, assuming that a fraction of the area reported into the first age class in 2002 was also affected by storm.
2. The widespread-storm disturbance event, was modelled assuming that only 50% of the existing living biomass is disturbed; also in this case, 46% of the aboveground merchantable biomass (i.e., about 23% of the total aboveground biomass) moves to the HWP pool and the remaining 54% (i.e., about 27% of the total aboveground biomass) moves to DOM pools. This event was applied into the same *i*-year of the stand replacing-storm but to a different area and it does not affect directly the age class distribution. While the stand replacing-storm was set based on the area (i.e., hectares), the effect of the widespread-storm (*DSL_W_i_* directly expressed as tons of carbon) was estimated as the difference between the total amount of biomass expected to be removed by direct salvage logging in the *i*-year (*DSL_T_i_*) and the amount of biomass already provided by the stand-replacing storm in the same year (*DSL_R_i_*):

${DSL\_W}_{i}={DSL\_T}_{i}-{DSL\_R}_{i}$ Eq. (6)

1. A further amount of harvest can be provided during the 3 years following the storm event, moving 60% of the stem snag biomass (coming from the widespread-storm) to the HWP pool. This salvage-logging “disturbance” has no effect on living biomass and it was set as a maximum amount of forest area affected by this event per each year, equal to 50,000 ha yr^-1^ (i.e., about 1/3 of the maximum forest area affected by the widespread-storm events in 2000 and 2007)

The storm occurred at the end of December 1999 was directly applied to the year 2000.

### Supplementary results

Figure 5 reports the amount of harvest provided by salvage of logging residues after the storms distinguished between direct removals on the area affected by widespread and stand-replacing storms and secondary salvage of logging residues after the disturbance events (see the Supplementary Material for further details).


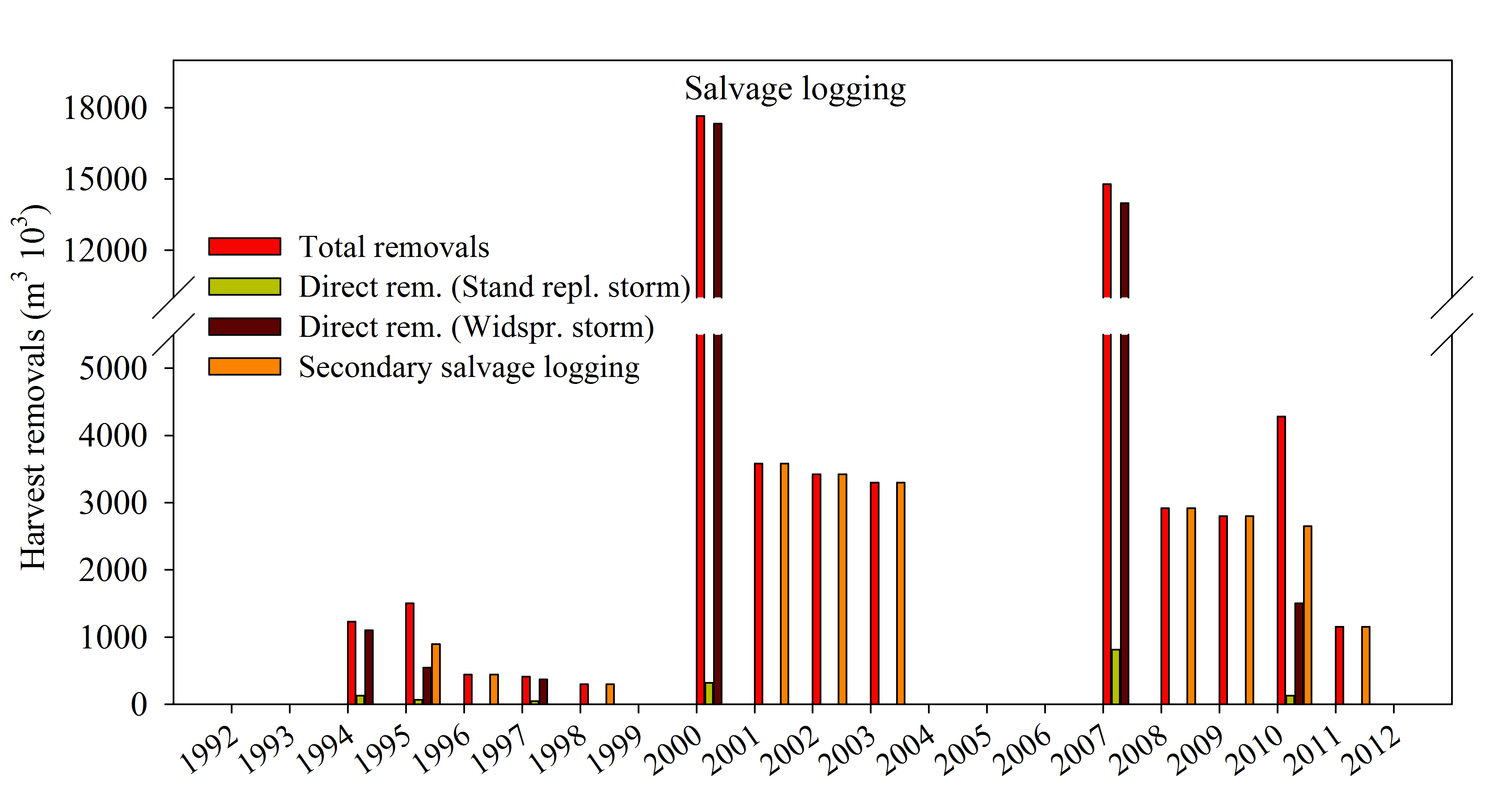


Figure 5: the figure reports the amount of harvest (in m^3^ 10^3^) provided by salvage of logging residues after the storm disturbances, distinguished as: total removals; direct removals on the area affected by stand-replacing disturbances; direct removals on the area affected by widespread disturbances; and secondary salvage of logging residues after the disturbance event.

Figure 6 reports the age class evolution provided by CBM model, compared with the original age class distribution reported by NFI, referred to 2002.


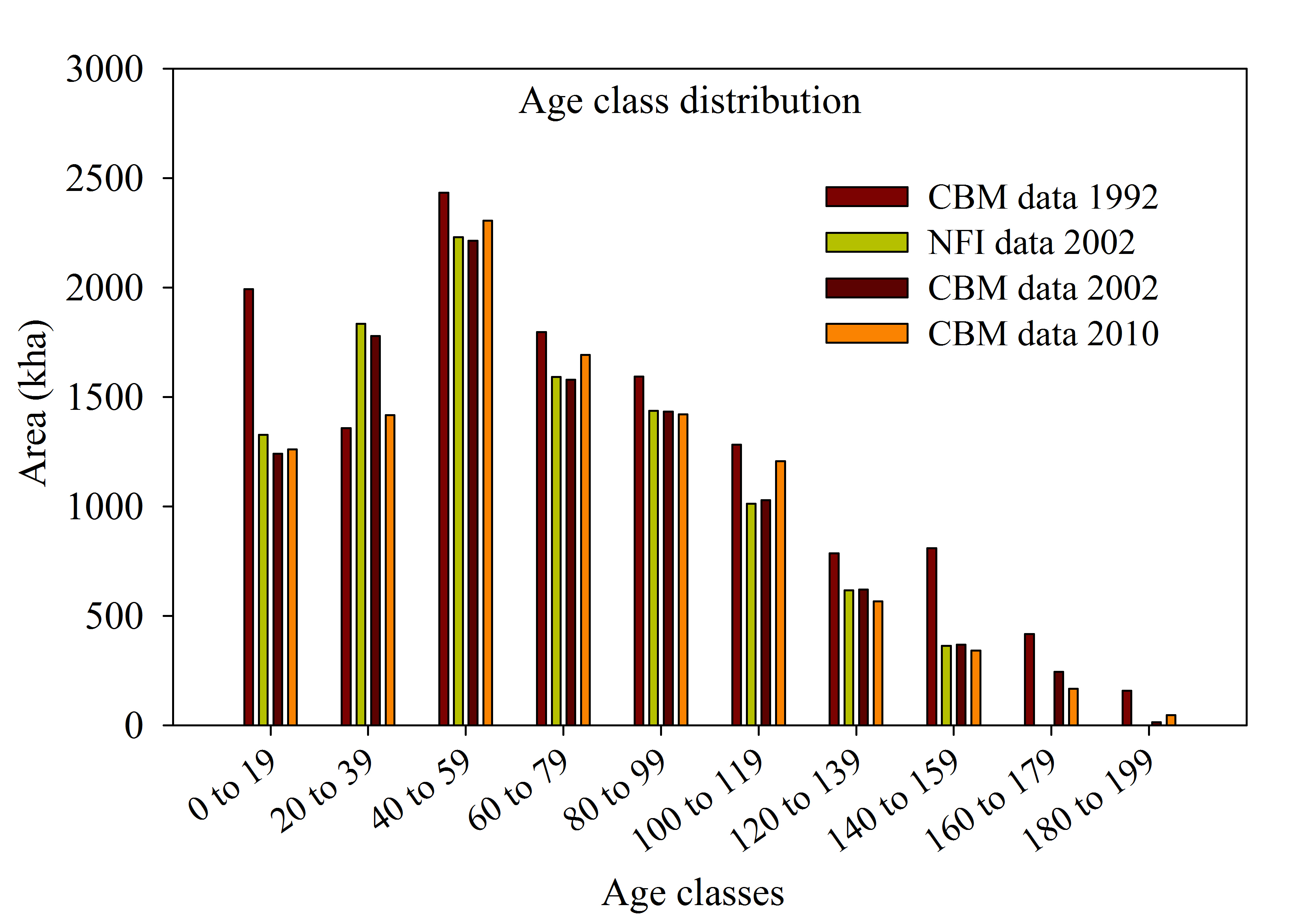


Figure 6: age class evolution provided by CBM model between 1992 and 2010, including the effect of deforestation; the original NFI age class distribution (assigned to 2002) is also reported.

## Lithuania

### Methodological assumptions

The analysis was based on the data collected by NFI 2004-2008 (referred to 2006; [15], [16]), scaled back to 1996. The total forest area, equal to 2,000 kha was corrected to account for the total amount of deforestation occurred until 1996 (i.e., about 53 ha yr^-1^). The main species reported by the NFI were grouped in 7 forest types (FTs, see Tab. 9), managed as high forests, with specific rotation lengths for each FT, based on the minimal age for final felling reported by the Report of the technical assessment of the forest management reference level submission of Lithuania submitted in 2011 ([17]).

| **Forest type**  **(main species)** | **FT Acronym** | **Min. rotation length (yrs)** |
| --- | --- | --- |
|  |  |  |
| *Alnus glutinosa* | **AG** | **60** |
| *Alnus incana* | **AI** | **30** |
| *Betula sp.* | **BT** | **60** |
| *Other Broadleaves(including Oaks and Ash)* | **OB** | **110** |
| *Picea abies* | **PA** | **70** |
| *Pinus sylvestris* | **PS** | **100** |
| *Poplar sp.* | **PT** | **40** |

Tab. 9: main species grouped by forest types and minimum rotation length applied by CBM model.

The main parameters defining the harvest criteria applied by CBM for Lithuania are reported on Tab. 10.

| **Silvicultural treatment** | **Criteria** | **Harvest share** |
| --- | --- | --- |
| 15% Commercial Thinnings | From 10-15 to 40-60 yrs depending by FTs | 8% |
| 30% Commercial Thinnings | From 10-60 to 40-125 yrs depending by FTs | 26% |
| Clearcut (90-95% commercial thinning) | Depending by species | 66% |

Tab. 10: main parameters defining the harvest criteria applied by CBM for Lithuania, including the age classes affected by each silvicultural treatment and the relative amount of harvest provided by each treatment (estimated as the average amount of harvest provided between 1996 and 2012).

Since no specific data on the biomass stock was available at national level, the same equations selected for Latvia (used to convert the volume to biomass and C content) were applied to Lithuania. Species-specific YTs were selected using the average volume and increment reported at national level. Since the NFI^[[4]](#footnote-4)^ ([16]) reports the gross annual increment, including the volume of dead trees by species and age classes, original NFI data were corrected to account for natural mortality and to estimate the net annual increment.

The effect of three different natural disturbances was considered:

1. Storms: based on the information reported by NIR ([18]), various storms damaged Lithuanian forests between 2000 -2005. According to data reported by the FORESTORMS database ([14]), the following information were collected (Tab. 11):

| **Model INPUT** | | **Model OUTPUT** | |
| --- | --- | --- | --- |
| **Year** | **Vol damaged**  **(M m^3^)** | **Vol affected by storm (M m^3^)** | **Area affected by storm (ha)** |
| **1996** |  |  |  |
| **1997** |  |  |  |
| **1998** |  |  |  |
| **1999** |  |  |  |
| **2000*** | 400,000 | 373,733 | 1,160 |
| **2001** |  |  |  |
| **2002** |  |  |  |
| **2003** |  |  |  |
| **2004** |  |  |  |
| **2005** | 1,000,000 | 1,028,814 | 2,904 |
| **2006** |  |  |  |
| **2007** | 300,000 | 307,401 | 870 |
| **2008** |  |  |  |
| **2009** |  |  |  |
| **2010** |  |  |  |
| **2011** |  |  |  |
| **2012** |  |  |  |
| * “Lothar”, Dec 1999, assumed as occurred in 2000 | | | |

Tab. 11: the table reports the volume damaged by the main storms, as reported by FORESTORM database (in million m^3^ reported as Model INPUT); the volume and area (in ha) directly affected by each disturbance event according to model’s assumptions (reported as Model OUTPUT).

We simulated these disturbances as “stand-replacing events”, affecting spruce forests^[[5]](#footnote-5)^ and moving 80% of the living biomass to snags pools, without any direct salvage of logging residues (i.e., this means that we also assumed that a fraction of the area reported in the first age class for spruce in 2006, was not directly clearcut but affected by storm).

Salvage of logging residues after natural disturbances, was simulated through specific events, moving biomass from stem snags pools to product pools. These events were further distinguished between two treatments, assuming that products were used as fuelwood or industrial roundwood and prioritizing, in some cases, the removals of harvest residues, from the stands having the highest stem snag carbon amount^[[6]](#footnote-6)^.

1. Fires: data on the amount of burned area (Figure 7) were directly taken from the NIR ([18]). Fire disturbances were simulated assuming that fire affect 50% of the living biomass, with salvage of 15% of logging residues.


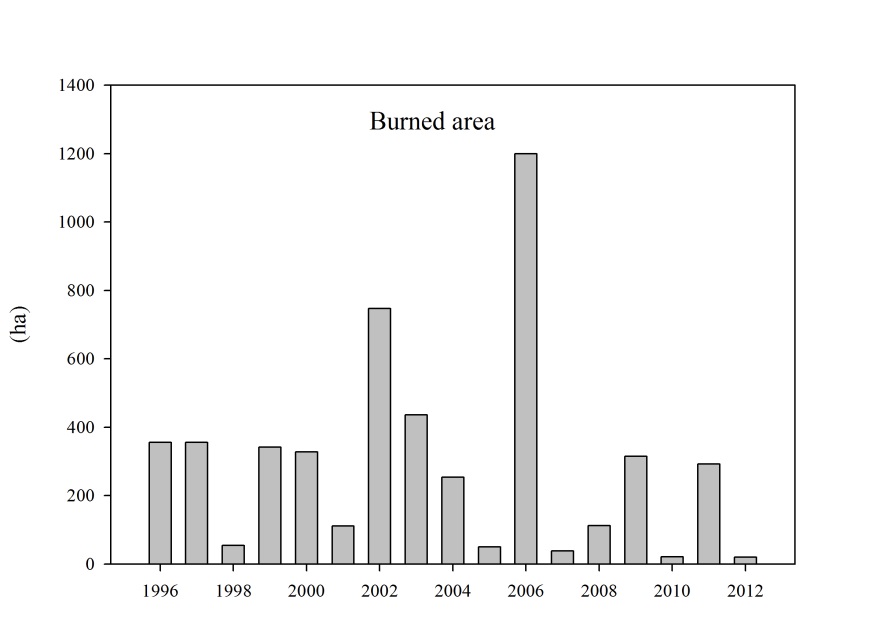


Figure 7: amount of area burned between 1996 and 2012 based on data reported by NIR ([18]).

1. Insects: according to NIR ([18]) a spruce dieback, caused by the bark beetle *Ips typographus* affected at least 13 million m^3^ of spruce between 1994 and 1997. Since no specific information about this attack is reported by the EFI database on Forest Disturbances in Europe ([19]), we first assumed that about 30,000 ha yr^-1^ of spruce were disturbed in 1996 and 1997 (with a mortality of 5% of the living biomass on the area affected by disturbance). Secondly, considering that insects attacks largely affected all coniferous species between 1994 and 1997 (according to DFDE database pines were also affected by other insects attacks from *Dendrolimus pini*), we assumed that these disturbances largely decreased the growth potential of coniferous species in 1996 and 1997. This was simulated applying a growth multiplier correction factor equal to 0.01 to spruce and pine forest area in 1996 and 1997.

As for storms, salvage of logging residues after these disturbances was simulated through specific events, moving biomass from stem snags pools to product pools. These events were further distinguished between two treatments, assuming that products were used as fuelwood or industrial roundwood.

The 2014 NIR ([18]) reports specific assumptions both on the living biomass and on the dead tree stems volumes, based on two specific studies performed at national level, which can be used to validate our model’s results. For this reason, further assumptions were considered to model the dead wood pool:

1. We distinguished coniferous and broadleaved FTs (assuming different Climatic Units with the same mean annual temperature and precipitation), in order to apply different stem annual turnover rates and snag fall rates for conifers and broadleaves.
2. Different stem annual turnover rates and snag fall rates were applied to the initialization process and during the model run.

The final values applied to DOM turnover parameters (Tab. 12) were based on a preliminary calibration (as suggested by [6]), taking into account the model output and the values provided by NIR ([18]).

| **CBM stage** | **Initialization Process** | | **Model run** | |
| --- | --- | --- | --- | --- |
| DOM Turnover parameters | Stem annual turnover rate | Stem snag fall rate | Stem annual turnover rate | Stem snag fall rate |
| Conifers | 0.001 | 0.028 | 0.003 | 0.009 |
| Broadleaves | 0.002 | 0.028 | 0.028 | 0.018 |

Tab. 12: CBM DOM turnover rates applied to Lithuania during the Initialization Process and the Model run, for coniferous and broadleaved species (further details on the meaning of these parameters are reported by [20])

## Poland

### Methodological assumptions

The analysis was based on the data collected by the available NFI referred to 1993 and provided by the EFISCEN database ([21]), therefore original data were not scaled back. The total forest area equal to 8,873 kha was decreased in order to account for the total amount of deforestation occurred until 1993 (i.e., about 540 ha yr^-1^). The main species reported by the NFI were grouped in 9 forest types, reported in Tab. 13. All species were managed as high forests. Specific rotation lengths were applied for each FT, according to the information reported by the Submission of information on forest management reference level of Poland ([22]).

| **Forest type**  **(main species)** | **Acronym** | **Min. rotation length (yrs)** |
| --- | --- | --- |
|  |  |  |
| *Abies alba* | AA | **120** |
| *Alnus sp. (we assumed that Black Alder was the main species)* | AG | **80** |
| *Betula sp.* | BT | **70** |
| *Fagus sylvatica* | FS | **100** |
| *Other broadleaves* | OB | **80** |
| *Picea abies* | PA | **80** |
| *Pinus sp.* | PS | **80** |
| *Populus sp.* | PT | **40** |
| *Quercus sp. (we assumed that Quercus robur was the main species)* | QR | **130** |

Tab. 13: main species grouped by forest types and minimum rotation length applied by CBM model.

The main parameters defining the harvest criteria applied by CBM for Poland are reported on Tab. 14

| **Silvicultural treatment** | **Criteria** | **Harvest share** |
| --- | --- | --- |
| 20% Commercial Thinnings | > 10 yrs. | 33% |
| 30% Commercial Thinnings | > 10 yrs. | 24% |
| Clearcut (95% commercial thinning) | Depending by species | 41% |
| Salvage logging after Nat. Disturbances | Depending by years | ≈ 1% |

Tab. 14: main parameters defining the harvest criteria applied by CBM for Poland, including the age classes affected by each silvicultural treatment and the relative amount of harvest provided by each treatment (estimated as the average amount of harvest provided between 1993 and 2012).

Since no specific data on biomass stock was available at national level, the equations selected for Germany and Latvia were applied to Poland.

Species-specific YTs were selected using the average volume and increment reported at national level. Since the NFI reports the gross annual increment (including the volume of dead trees) by species and age classes, original NFI data were corrected to account for natural mortality.

The effect of two main storms was considered according to the following assumptions:

1. Based on the information reported by the FORESTORMS ([14]) database, the following storms affected Poland’s forests during the last years (Tab. 15).

| **Year** | **Primary damage (Mm3)** |
| --- | --- |
| 1999 | 2.0 |
| 2007 | 3.0 |

Tab. 15: amount of merchantable volume (in million m^3^) damaged by storms in Poland, as reported by the FORESTORMS database.

1. The volume damaged by each storm was distributed between each FT according to its proportion in the total forest area and converted to tons of C using the wood density.
2. The disturbance event was simulated as a widespread –storm (i.e., not stand replacing), affecting 15% of the living biomass, with a direct salvage of logging residues. This one was simulated moving 10% of the merchantable biomass to the product pool and the remaining 5% to DOM.

### Supplementary results

Figure 8 reports the age class evolution provided by CBM model between 1993 and 2012. In this case no comparison with the original NFI data was reported, because the NFI input data refer to 1993.


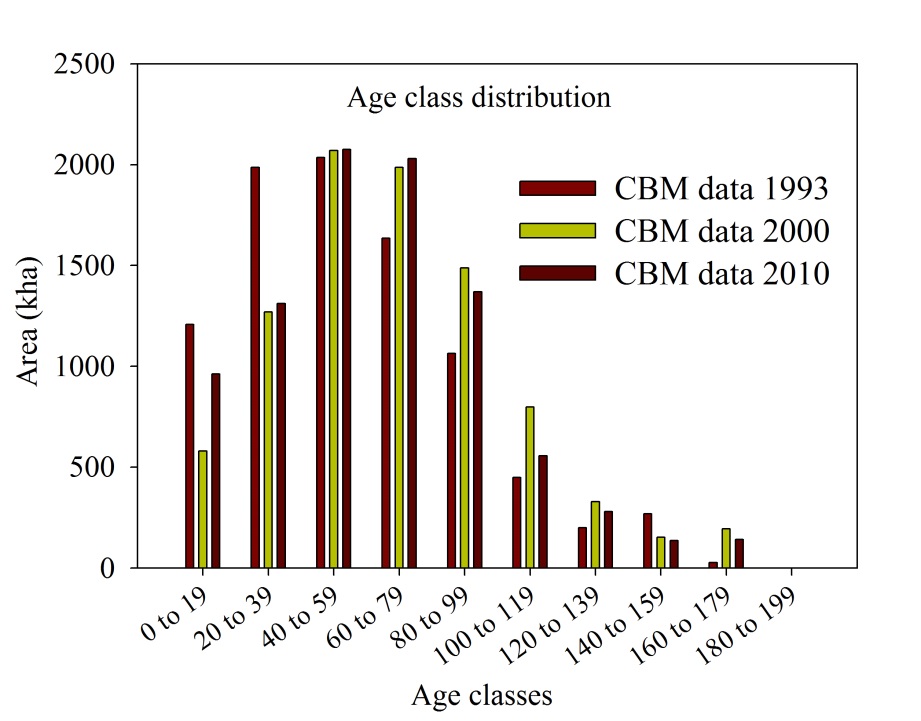


Figure 8: age class evolution provided by CBM model between 1993 and 2012, including the effect of deforestation.

## Portugal

### Methodological assumptions

The analysis was based on the data reported by the 2005 NFI, as provided by the EFISCEN database ([21]), reporting a total forest area equal to 3,169 kha. The original age class distribution was scaled back to 1995 and to a forest area equal to 3,614 kha (i.e., the FM area referred to 1990 equal to 3,700 kha, minus 5 times the annual rate of deforestation reported by Portugal, equal to 14.2 kha yr^-1^). The total forest area reported by NFI was further scaled by age classes and species according to the additional information reported by the Submission for forest management reference level of Portugal ([17]).The main species reported by NFI were grouped in 8 forest types (Tab. 16). All species, except *Eucaliptus sp.*, were managed as high forests. We assumed that the area reported as “irregular forests” was managed as uneven-aged forest. Specific rotation lengths were applied for each even-aged FT, according to the values reported by literature (Tab. 16).

| **Forest type**  **(main species)** | **Acronym** | **MT** | **Min. rotation length applied to even-aged (yrs)** |
| --- | --- | --- | --- |
|  |  | **MS** |  |
| *Pinus pinaster* | PM | **H E /U** | **35** |
| *Pinus pinea* | PP | **H E/U** | **95** |
| *Other conifers* | OC | **H E/U** | **65** |
| *Eucaliptus spp.* | EG | **C E /U** | **8-20** |
| *Other broadleaves* | OB | **H E/U** | **65** |
| *Quercus rotundifolia* | QH | **H E/U** | **105** |
| *Quercus spp.* | QR | **H E/U** | **65** |
| *Quercus suber* | QS | **H E/U** | **105** |

Tab. 16: main species grouped by forest types and minimum rotation length applied by CBM model to the even-aged forests. The table also reports the management type (H, for high forests and C for coppices) and the management strategy (E, for even-aged forests and U for uneven-aged forests) applied to each FT.

The main parameters defining the harvest criteria applied by CBM for Portugal are reported on Tab. 17.

| **Silvicultural treatment** | **Harvest criteria** | **Harvest share^1^** |
| --- | --- | --- |
| 20% Commercial Thinnings | **HE**: > 20 yrs. | <1% |
| 25% Commercial Thinnings | **HE**: > 15 yrs.  **HU**: minimum every 7 years | 14% |
| 30% Commercial Thinnings | **C**: > 10 yrs.  **HU**: minimum every 12 years | 9% |
| 35% Commercial Thinnings | **HU**: minimum every 6 years | 5% |
| Clearcut - 95% commercial thinning | **HE/C**: Depending on species | 56% ^1^ |
| Salvage logging on burned area (Min 15% of merchantable biomass) | | 15% ^1^ |
| 1: depending by year, according to the amount of harvest provided by burned area | | |

Tab. 17: main parameters defining the harvest criteria applied by CBM for Portugal, including the age classes affected by each silvicultural treatment (or the minimum cutting cycle for uneven aged forests) and the Management strategy (MS) where each treatment was applied (HE=evenaged high forests, HU=unevenaged high forests, C=coppices) the relative share of harvest provided by each treatment (average for the historical period).

Country-specific equations were selected using the volume and the aboveground biomass data provided by literature^[[7]](#footnote-7)^. The average aboveground total biomass by species was compared with the values provided by the original set of equations reported by [4] for Quebec ([6]). The equations selected for Portugal are reported Tab. 18.

| **FT** | **Species selected by default CBM database** |
| --- | --- |
| PM | Red pine (*P. resinosa*) |
| PP | Jack Pine (*P. banksiana*) |
| OC | Red pine (*P. resinosa*) |
| EG | Red pine (*P. resinosa*) |
| OB | White elm (*U. americana*) |
| QH | White elm (*U. americana*) |
| QR | White elm (*U. americana*) |
| QS | Green ash (*F. pennsylvanica var. subintegerrima*) |

Tab. 18: association between the forest types and the default species provided by the original CBM database.

Tab. 19 reports the percentage difference between the average aboveground total biomass estimated by the selected equations and the country-specific values of biomass.

| **FT** | **Mean Δ** | **St dev.** |
| --- | --- | --- |
| EG | 24.96 | 12.43 |
| PM | 45.27 | 10.97 |
| PP | 21.67 | 27.22 |
| QH | -5.87 | 5.34 |
| QR | 20.80 | 3.68 |
| QS | 13.90 | 26.74 |

Tab. 19: the table reports the mean percentage difference and the standard deviation between the average aboveground total biomass estimated by the selected CBM equations and the country-specific biomass values (the mean and the standard deviations were estimated considering the values reported by FT and age class)

Species-specific YTs were selected using the average volume and increment reported by NFI at regional level, applying specific correction factors (reported by Tab. 20) based on the values of increment reported by the Submission of information on forest management reference levels by Portugal ([17]). Further correction factors were also applied to the original data of volume (referred to “pure stands”) to account for the presence of other dominated tree species in each stands. These Mixed Correction Factors (Mix CF reported in Tab. 20) were estimated as the ratio between the average volume of the dominant species and the total volume reported by the NFI for each FT (see Table 7-12, [23])

|  | **Increment (m^3^ ha^-1^ yr^-1^)** | | **Increment CF** | **Mix CF (volume)** |
| --- | --- | --- | --- | --- |
| **FT** | **NFI** | **Submission** |  |  |
| EG | 3.8 | 9.5 | **2.49** | **1.14** |
| PM | 2.4 | 5.6 | **2.31** | **1.11** |
| PP | 3.6 | 5.6 | **1.57** | **1.44** |
| OC | 2.1 | 5 | **2.42** | **1.07** |
| QS | 1.1 | 0.5 | **0.45** | **1.17** |
| QH | 0.5 | 0.5 | **1.08** | **1.25** |
| QR | 1.4 | 2.9 | **2.01** | **1.66** |
| OB | 4.9 | 2.9 | **0.59** | **1.88** |

Tab. 20: the correction factor (CF) applied to the increment values reported (region by region and species by species) by the original NFI data (reported in the first column) was estimated as the ratio between the NFI data and data provided by the country submission.

According to the information reported by the NIR ([23]), Eucalyptus plantations are harvested in a period of 12 years (i.e., according to KP, before that the 20 yrs. conversion period from AR to FM is completed)^[[8]](#footnote-8)^. Therefore, for Portugal, we assumed that a fraction of the industrial roundwood broadleaves demand (*IRW_B_*) is directly provided by AR activities (*IRW_AR_*), on Eucalyptus plantations, managed through clearcuts, with a minimum rotation period equal to 12 yrs. (see [24] for further details on the application of CBM for AR activities). The amount of IRW broadleaves provided by FM was therefore estimated as the difference between the total *IRW_B_* and *IRW_AR_*.

Due to the major importance of forest fires in Portugal, these were taken into consideration as the main natural disturbance in the country. Figure 9 shows the historical area affected by fires based on data reported by the CRF tables for 2014 ([2]).


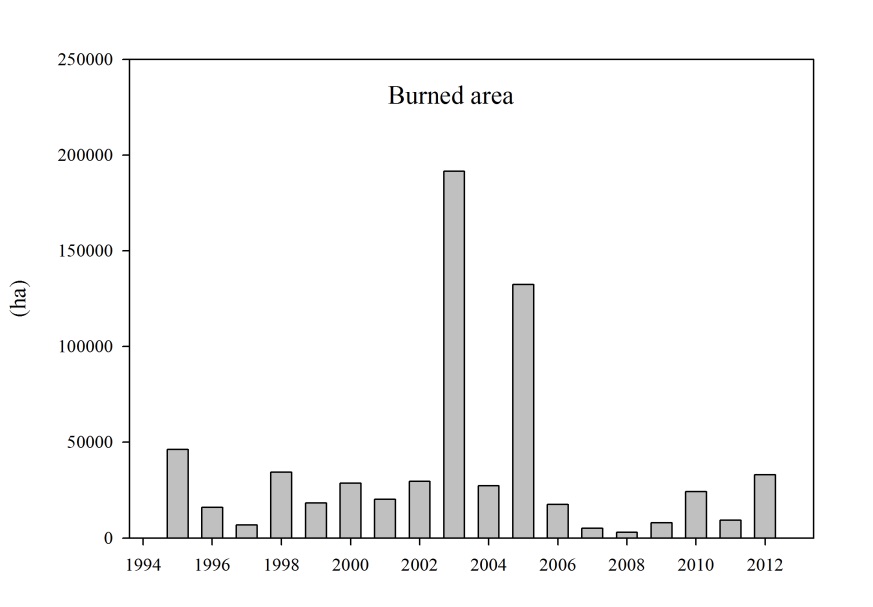


Figure 9: amount of area burned between 1995 and 2012 based on data reported by 2014 CRF tables

The total forest area affected by fires was first distributed between the main FTs (see Tab. 21), according to the additional information reported by the Submission for FMRL ([17]^[[9]](#footnote-9)^) on the total amount of burned area (by species) referred to 2013.

| Forest types | Fire distribution applied to the total burned area | Proportion of burned area affected by Stand replacing disturbance event (*p_SR_*) |
| --- | --- | --- |
| PM | 0.32 | 0.25 |
| QS | 0.07 | 0.2 |
| EG | 0.40 | 0.5 |
| QH | 0.03 | 0 |
| QR | 0.12 | 0 |
| OB | 0.04 | 0.1 |
| PP | 0.02 | 0.4 |
| OC | 0.01 | 0 |

Tab. 21: the first column reports, for each FT, the relative proportion of the total burned area affected (each year) by fire disturbances. The second column reports the proportion of burned area affected by Stand replacing disturbance event (*p_SR_*).

According to the information reported by the country a fraction of the total harvest demand comes from the amount of merchantable wood removed by the area affected by fires (i.e., salvage of logging residues). We defined two possible fire-disturbances:

1. *Fire Stand Replacing*: assuming that on the area affected by fire, a complete salvage of logging residues (i.e., on 100% of the merchantable biomass) was provided and the stand moves to the age class 0 after the disturbance event. In this case all the living biomass components not moved to the product pool will move to the DOM pool.
2. *Fire Not-Stand Replacing*: assuming that the forest area was only partially affected by fire (i.e., the stand maintains the current age class after the disturbance event and about 25% of the living biomass was burned). In this case, based on the mortality rates reported by NIR (See Table 7-27, [23]) we defined the average mortality rate of broadleaves (26%) and conifers (63%). We assumed that half of the dead merchantable biomass (i.e., 13% for broadleaves and 31.5% for conifers) was harvested (i.e., moved to the products pool through salvage of logging residues) while the remaining merchantable biomass (such as 26% and 63% of the other living biomass components) moves to the DOM pool.

In order to define the amount of burned area affected by these disturbance events, we first considered the total amount of forest area reported by the original NFI distribution into the first age class (i.e., with age < 10 yrs., therefore potentially affected by a stand replacing disturbance event during the previous 10 yrs.). We defined for each FT a constant proportion of the total burned area potentially affected by stand-replacing disturbance event (*p_SR_*).The remaining area (i.e., 1-*p_SR_*) will be affected by a not-stand-replacing disturbance event. The area affected by not-stand-replacing disturbances was therefore estimated as the difference between the total area affected by fire for each species and year and the stand-replacing area.

### Supplementary results

Figure 10 reports the direct fire emissions (in Gg CO_2Eq._) estimated by CBM, further compared with the emissions estimated by the country.


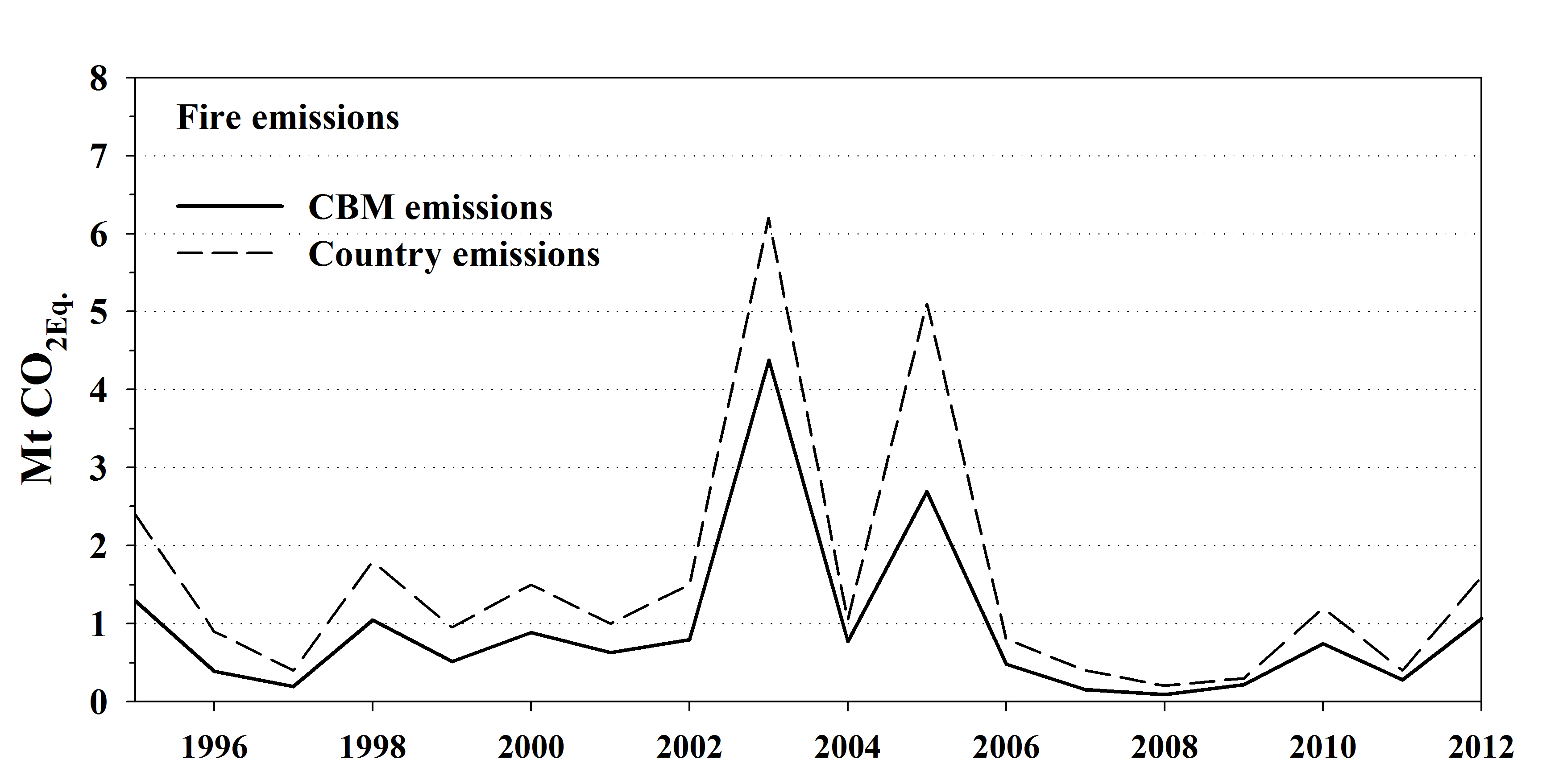


Figure 10: comparison between the direct fire emissions (in Gg CO_2Eq._) estimated by CBM and the emissions estimated by the country.

Figure 11 reports the age class evolution provided by CBM model between 1995 and 2012, compared with the original age class distribution reported by NFI for 2005.


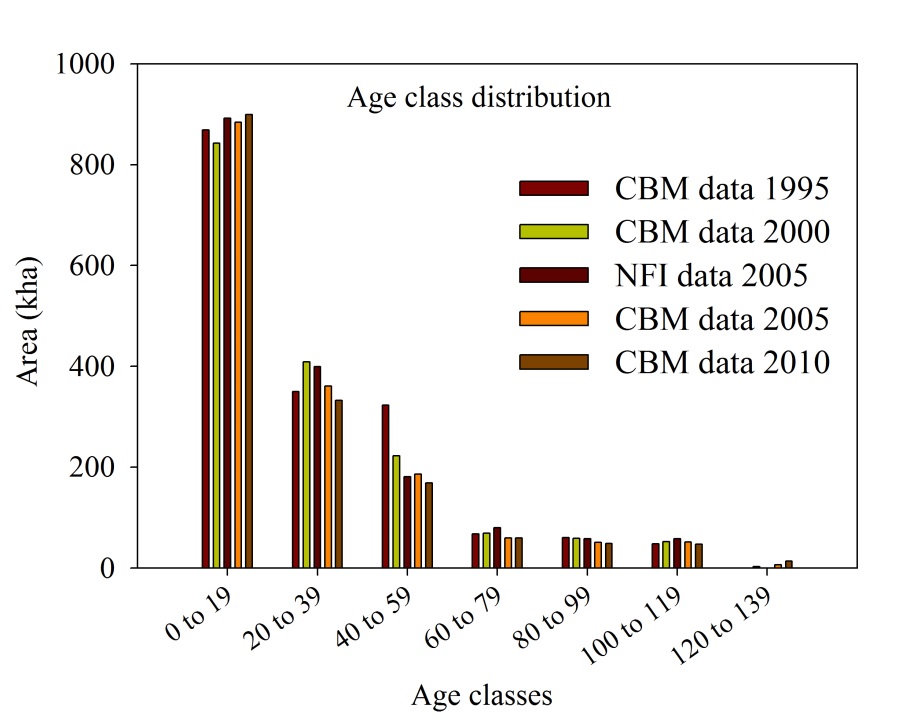


Figure 11: age class evolution provided by CBM model between 1995 and 2012 (only including the even-aged forests); the original NFI age class distribution (assigned to 2005) is also reported. Due to the effect of deforestation, the total forest area is decreasing.

## SUPPLEMENTARY REFERNCES

1. Austria, NFI web site. URL(last access March 2015): <http://web.bfw.ac.at/i7/oewi.oewi0002>
2. KP CRF tables, 2014. URL (last access March 2015): <http://unfccc.int/national_reports/annex_i_ghg_inventories/national_inventories_submissions/items/8108.php>
3. Austria. Austria’s National Inventory Report 2014 and CRF tables. URL (last access March 2015): <http://unfccc.int/national_reports/annex_i_ghg_inventories/national_inventories_submissions/items/8108.php>
4. Boudewyn P, Song X, Magnussen S, Gillis MD. Model-based, Volume-to-Biomass Conversion for Forested and Vegetated Land in Canada. Canadian Forest Service, Victoria, Canada, 2007 (Inf. Rep. BC-X-411). URL (last access, March 2015): <http://cfs.nrcan.gc.ca/publications/?id=27434>
5. Kurz WA, Dymond CC, White TM, Stinson G, Shaw CH, Rampley G., Smyth C, Simpson B.N., Neilson E., Trofymow JA, Metsaranta J, Apps MJ. CBM-CFS3: A model of carbon-dynamics in forestry and land-use change implementing IPCC standards. Ecol. Model., 2009, 220, 480-504.
6. Pilli R, Grassi G, Kurz WA, Smyth CE, Bluydea V. Application of the CBM-CFS3 model to estimate Italy’s forest carbon budget, 1995 to 2020. Ecol. Modell., 2013, 266: 144-171.
7. Gschwantner T, Gabler K, Schadauer K, Weiss P, In: Tompoo E, Gschwantner T, Lawrence M, McRoberts RE. National Forest Inventories. Pathways for Common Reporting. Springer, Heidelberg, Dordrecht, London, New York, 2010.
8. Tomter S M, Gasparini P, Gschwantner T, Hennig P, Kulbokas G, Kuliešis A, Polley H, Robert N, Rondeux J, Tabacchi G, Tompoo E. Establishing Bridging Functions for Harmonizing Growing Stock Estimates: Examples from European National Forest Inventories. Forest Science, 2012, 58: 224-235.
9. Foglar-Deinhardstein A, Hangler J, Prem J (eds.) Sustainable Forest Management in Austria – Austrian Forest Report, 2004 and 2008. Republic of Austria, Federal Ministry of Agriculture, Forestry, Environment and Water Management, Vienna. URL (last access March 2015): [www.lebensministerium.at](http://www.lebensministerium.at)
10. BMELV, Bundesministerium für Ernhärung, Landwirtschaft und Verbraucherschutz, 2006. The Second National Forest Inventory – NFI2. Results. Federal Ministry of Food, Agriculture and Consumer Protection, Wien.
11. Zianis D, Muukkonen P, Mäkipää R, Mencuccini M. Biomass and stem volume equations for tree species in Europe. Silva Fennica, 2005, 4.
12. Wirth C, Schumacher J, Schulze ED. Generic biomass functions for Norway spruce in Central Europe. A meta-analysis approach toward prediction and uncertainty estimation. Tree Physiology, 2004, 24, 121-139.
13. Germany. National Inventory Report for the German Greenhouse Gas Inventory 1990-2012. Federal Environment Agency, 2014. URL (last access March 2015): <http://unfccc.int/national_reports/annex_i_ghg_inventories/national_inventories_submissions/items/8108.php>
14. Goodale CL, Apps MJ, Birdsey RA, Field CB, Heath LS, Houghton RA, Jenkins JC, Kohlmaier GH, Kurz WA, Liu SR, Nabuurs GJ, Nilsson S, and Shvidenko AZ. Forest carbon sinks in the Northern Hemisphere. Ecological Applications, 2002, 12: 891–899.
15. Kuliešis A and Kulbokas G. Lithuanian National Forest Inventory 2004-2008. Forest Resources and their dynamic. Ministry of Environment, Stat Forest Survey Service, Kaunas, 2009a.
16. Kuliešis A and Kulbokas G. Forest Statistics. Ministry of Environment, Stat Forest Survey Service, Kaunas, 2009b.
17. AWG-KP, Ad Hoc Working Group on Further Commitments for Annex I Countries under Kyoto Protocol, 2011. Submissions for Forest Management Reference Level, 2011. URL (last access March 2015): <http://unfccc.int/bodies/awg-kp/items/5896.php>
18. Lithuania, 2014. Lithuania’s National Inventory Report 2014. URL (last access March 2015): <http://unfccc.int/national_reports/annex_i_ghg_inventories/national_inventories_submissions/items/8108.php>
19. DFDE, Forest Disturbances in Europe, EFI Database: URL (last access March 2015): <http://dataservices.efi.int/dfde>
20. Kull S, Kurz WA, Rampley G, Morken S, Metsaranta ET, Neilson ET. Operational-Scale Carbon Budget Model of the Canadian Forest Sector (CBM-CFS3) Version 1.2: User’s Guide. Canadian Forest Service, Northern Forestry Centre, 2011.
21. EFISCEN database, URL (last access March 2014): <http://dataservices.efi.int/authenticate.php>
22. Poland, 2014. Poland’s National Inventory Report 2014. URL (last access March 2015): <http://unfccc.int/national_reports/annex_i_ghg_inventories/national_inventories_submissions/items/8108.php>
23. Portugal, 2014. Portuguese National Inventory Report on Greenhouse Gases, 1990-2012. URL (last access March 2015): <http://unfccc.int/national_reports/annex_i_ghg_inventories/national_inventories_submissions/items/8108.php>
24. Pilli R, Grassi G, Moris JV, Kurz WA, 2014. Assessing the carbon sink of afforestation with the Carbon Budget Model at the country level: an example for Italy. iForest, 2014b, 8: 410-421.

1. Industrial roundwood [↑](#footnote-ref-1)
2. Fuelwood [↑](#footnote-ref-2)
3. As suggested by the CBM’s User Guide, the Sort type of this disturbance event was modified (Sort type 7) in the project database file. [↑](#footnote-ref-3)
4. Lithuanian national forest Inventory 2004-2008. Forest resources and their dynamic. Forest Statistics ([15]). [↑](#footnote-ref-4)
5. To simplify the model assumptions (above all for reconstructing the age class distribution before 2006), we assumed that the effect of storm was concentrated between the age classes 70 – 80 yrs., i.e. the average age of spruce forests in Lithuania. [↑](#footnote-ref-5)
6. As suggested by the CBM’s User Guide, the Sort type of this disturbance event was modified (Sort type 7) in the project database file. [↑](#footnote-ref-6)
7. Portuguese National Forest Authority, 2010: <http://www.icnf.pt/portal/florestas/ifn> [↑](#footnote-ref-7)
8. NIR Portugal, 2014, pag. 7-35 [↑](#footnote-ref-8)
9. See Table 14 reported by the Portugal’s Submission for FMRL [17]. [↑](#footnote-ref-9)
